# Association Between Socioeconomic Status and In Utero Fetal Brain Development

**DOI:** 10.1001/jamanetworkopen.2021.3526

**Published:** 2021-03-29

**Authors:** Yuan-Chiao Lu, Kushal Kapse, Nicole Andersen, Jessica Quistorff, Catherine Lopez, Andrea Fry, Jenhao Cheng, Nickie Andescavage, Yao Wu, Kristina Espinosa, Gilbert Vezina, Adre du Plessis, Catherine Limperopoulos

**Affiliations:** 1 Developing Brain Institute, Children's National Hospital, Washington, District of Columbia; 2 Department of Quality and Patient Safety, Children's National Hospital, Washington, District of Columbia; 3 Department of Pediatrics, School of Medicine and Health Sciences, George Washington University, Washington, District of Columbia; 4 Prenatal Pediatrics Institute, Children's National Hospital, Washington, District of Columbia

## Abstract

**Importance:**

Children raised in settings with lower parental socioeconomic status are at increased risk for neuropsychological disorders. However, to date, the association between socioeconomic status and fetal brain development remains poorly understood.

**Objective:**

To determine the association between parental socioeconomic status and in vivo fetal brain growth and cerebral cortical development using advanced, 3-dimensional fetal magnetic resonance imaging.

**Design, Setting, and Participants:**

This cohort study of fetal brain development enrolled 144 healthy pregnant women from 2 low-risk community obstetrical hospitals from 2012 through 2019 in the District of Columbia. Included women had a prenatal history without complications that included recommended screening laboratory and ultrasound studies. Exclusion criteria were multiple gestation pregnancy, known or suspected congenital infection, dysmorphic features of the fetus, and documented chromosomal abnormalities. T2-weighted fetal brain magnetic resonance images were acquired. Each pregnant woman was scanned at up to 2 points in the fetal period. Data were analyzed from June through November 2020.

**Exposures:**

Parental education level and occupation status were documented.

**Main Outcomes and Measures:**

Regional fetal brain tissue volume (for cortical gray matter, white matter, cerebellum, deep gray matter, and brainstem) and cerebral cortical features (ie, lobe volume, local gyrification index, and sulcal depth) in the frontal, parietal, temporal, and occipital lobes were calculated.

**Results:**

Fetal brain magnetic resonance imaging studies were performed among 144 pregnant women (median [interquartile range] age, 32.5 [27.0-36.1] years) with gestational age from 24.0 to 39.4 weeks; 75 fetuses (52.1%) were male, and 69 fetuses (47.9%) were female. Higher parental education level was associated with significantly increased volume in the fetal white matter (mothers: β, 2.86; 95% CI, 1.26 to 4.45; *P* = .001; fathers: β, 2.39; 95% CI, 0.97 to 3.81; *P* = .001), deep gray matter (mothers: β, 0.16; 95% CI, 0.002 to 0.32; *P* = .048; fathers: β, 0.16; 95% CI, 0.02 to 0.31; *P* = .02), and brainstem (mothers: β, 0.06; 95% CI, 0.02 to 0.10; *P* = .01; fathers: β, 0.04; 95% CI, 0.004 to 0.08; *P* = .03). Higher maternal occupation status was associated with significantly increased volume in the fetal white matter (β, 2.07; 95% CI, 0.88 to 3.26; *P* = .001), cerebellum (β, 0.17; 95% CI, 0.04 to 0.29; *P* = .01), and brainstem (β, 0.03; 95% CI, 0.001 to 0.07; *P* = .04), and higher paternal occupation status was associated with significantly increased white matter volume (β, 1.98; 95% CI, 0.71 to 3.25; *P* < .01). However, higher socioeconomic status was associated with significantly decreased fetal cortical gray matter volume (mothers: β, −0.11; 95% CI, −0.18 to −0.03; *P* = .01; fathers: β, −0.10; 95% CI, −0.18 to −0.03; *P* = .01). Higher parental socioeconomic status was associated with increased volumes of 3 brain lobes of white matter: frontal lobe (mothers: β, 0.07; 95% CI, 0.02 to 0.13; *P* = .01; fathers: β, 0.06; 95% CI, 0.01 to 0.11; *P* = .03), parietal lobe (mothers: β, 0.07; 95% CI, 0.03 to 0.11; *P* < .001; fathers: β, 0.06; 95% CI, 0.03 to 0.10; *P* = .001), and temporal lobe (mothers: β, 0.04; 95% CI, 0.02 to 0.07; *P* < .001; fathers: β, 0.04; 95% CI, 0.02 to 0.07; *P* < .001), and maternal SES score was associated with significantly decreased volume in the occipital lobe (β, 0.02; 95% CI, 0.002 to 0.04; *P* = .03). Higher parental socioeconomic status was associated with decreased cortical local gyrification index (for example, for the frontal lobe, mothers: β, −1.1; 95% CI, −1.9 to −0.3; *P* = .01; fathers: β, −0.8; 95% CI, −1.6 to −0.1; *P* = .03) and sulcal depth, except for the frontal lobe (for example, for the parietal lobe, mothers: β, −9.5; 95% CI, −13.8 to −5.3; *P* < .001; fathers: β, −8.7; 95% CI, −13.0 to −4.4; *P* < .001).

**Conclusions and Relevance:**

This cohort study found an association between parental socioeconomic status and altered in vivo fetal neurodevelopment. While being born and raised in a lower socioeconomic status setting is associated with poorer neuropsychological, educational, and socioeconomic outcomes in children, these findings suggest that altered prenatal programming may be associated with these outcomes and that future targeted prenatal interventions may be needed.

## Key Points

**Question:**

Is parental socioeconomic status associated with fetal brain development?

**Findings:**

In this cohort study of 144 healthy pregnant women and their fetuses, low parental socioeconomic status was associated with decreased regional fetal brain tissue volume and increased brain gyrification.

**Meaning:**

This cohort study found that parental socioeconomic status was associated with altered fetal brain morphology as measured by quantitative magnetic resonance imaging techniques, which may serve as an early biomarker associated with childhood neurodevelopmental disorders.

## Teaser

This cohort study examines the association between parental socioeconomic status measures and brain volume and cortical features among fetuses.

## Introduction

Socioeconomic status (SES) is the social standing or class of an individual or group.^1,2,3^ It is a complex construct that encompasses household income, material resources, education, and occupation. Among individuals with lower SES backgrounds, SES-associated variability in children’s experiences has been associated with not only adverse cognitive and social-emotional development throughout childhood and adolescence, but also higher rates of depression, anxiety, and attention and conduct disorders.^4,5^ Moreover, a 1998 study^6^ found that a child's brain development may vary by SES background, typically indexed by family income and parental education. Available evidence suggests that lower SES is associated with decreased regional brain growth in infants,^7,8,9^ particularly in the frontal and parietal lobe volumes, and decreased cortical gray matter (CGM) and deep gray matter (DGM) volumes in older children and adolescents.^2,10,11,12,13,14,15,16^ These findings of lower SES and altered brain structure have been associated with impaired neurocognitive function.^1,10,17^ While the association of SES measures, including family income, parental occupation, and parental education, with brain development has been widely investigated, many questions remain unanswered. These include questions about when the onset and timing of brain development diverges in populations that are at high risk for adverse developmental outcomes and have SES-associated disparities and what, if any, association SES has with fetal cortical maturation. The ability to interrogate early brain development, beginning in the fetal period, may help analysts and policy makers identify levels of intervention and entry points for action to support and optimize well-being of populations at high risk for adverse developmental outcomes.

The goal of this study was to determine the association between SES and fetal brain development using in vivo magnetic resonance imaging (MRI) and 3-dimensional computational brain models among healthy fetuses. We hypothesized that SES would be associated with fetal brain volumetric growth and cerebral cortical development in healthy fetuses. Notably, this study allowed us to investigate the association between SES and emerging fetal brain morphologic characteristics and gyrification without the confounding associations of well-established postnatal environmental variables.^7,8,9,10,15^

## Methods

This cohort study was approved by the Children’s National Hospital institutional review board, and written informed consent was obtained from all participants. We followed the Strengthening the Reporting of Observational Studies in Epidemiology (STROBE) reporting guideline.

### Study Participants

We prospectively enrolled 144 healthy pregnant women in a longitudinal fetal brain MRI study from 2 low-risk obstetrical community hospitals from 2012 through 2019 in the District of Columbia. Healthy pregnant volunteers were included if they had a prenatal history without complications that included recommend screening laboratory and ultrasound studies. Exclusion criteria were multiple gestation pregnancy, known or suspected congenital infection, syndromic features or dysgenetic lesions of the fetus, and documented chromosomal abnormalities. We also excluded pregnant women with any maternal contraindication to MRI. Enrolled fetuses with postnatal confirmation of a genetic syndrome, significant extracardiac anomalies, or structural (eg, encephaloclastic or dysgenetic) brain abnormalities on fetal MRI were excluded from the study. Participating women self-identified their race/ethnicity from a list of options defined by the investigators.

### MRI Data Acquisition

Multiplane, multiphase single-shot fast spin echo (SSFSE) T2-weighted images for fetal brain were acquired on a 1.5 Tesla GE Discovery MR450 scanner (GE Healthcare) using an 8-channel surface receiver coil (USA Instruments) (eFigure 1 in the Supplement). The acquisition parameters were used as follows: echo time, 160 ms; repetition time, 1100 ms; field of view, 320 × 320 mm^2^; dimension; 256 × 256; slice thickness, 2 mm; and 50 to 70 consecutive slices for full fetal brain coverage in the axial, coronal, and sagittal planes for a final in-plane resolution of 1.25 × 1.25 mm^2^. Each fetus was scanned at up to 2 points in the fetal period.

### SES Measures

Parental education and occupation were documented at the time of each MRI visit. We acquired SES data from parents’ responses to a sociodemographic questionnaire, which is an adjusted version of the Hollingshead Two Factor Index of Social Position.^18^ The level of education and occupational status were coded from 1 to 5 (ie, lowest to highest). Maternal and paternal SES scores were derived by summing the occupation status, which had a weight of 7, and the education status, which had a weight of 4.^10^ The resulting SES score ranged from 11 (ie, unemployment and no formal education beyond obligatory schooling) to 55 (ie, higher executive with postcollege education).

### Image Processing

Fetal brain T2-weighted multiplane images were first motion corrected using the slice-to-volume registration method.^19^ This procedure reduced interslice motion artifacts and provided images with enhanced contrast and resolution, along with coherent anatomic boundaries in 3-dimensional space. Automatic segmentation was applied using the Developing Brain Region Annotation with Expectation-Maximization (Draw-EM) algorithm.^20,21^ Two sets of tissue labels were generated from Draw-EM: the segmentation file with 9 labels^20^ and the parcellation file with 50 labels.^21^ Manual correction of tissue labels of the segmentation and parcellation files was performed by a trained research team member (K.K.) with more than 5 years of experience in fetal brain segmentation using Insight Segmentation and Registration Toolkit-Snake Automatic Partitioning (University of North Carolina).^22^ From among 184 scans taken, a second trained examiner (N.R.A.) randomly chose and segmented 35 scans (19.0%). Inter-rater reliabilities using intraclass correlation coefficient for all measured regions were higher than 0.95.

We extracted 10 connected brain regions from segmentation and parcellation files (eFigure 2 and eFigure 3 in the Supplement). The frontal, parietal, temporal, and occipital lobes; anterior and posterior cingulate gyrus; insula; and corpus callosum were extracted from the parcellation file, and the DGM and ventricles were extracted from the segmentation file. The connected brain regions were imported to BrainSuite version 18a (University of California, Los Angeles) to generate 3-dimensional surface mesh models.^23^ Each mesh model included a set of 3-dimensional coordinates for the surface vertices and a set of triangular meshes (faces). Every surface vertex was associated with 1 of the connected brain regions.

### Fetal Brain Volumes

The volumes of the brain tissues from the segmentation file were determined based on the voxel sizes of the images. The tissues included the CGM, white matter (WM), DGM, cerebellum, and brainstem (eFigure 2 in the Supplement).

### Cortical Folding

Gyrification or fetal brain cortical folding is the process by which the brain undergoes changes in surface morphologic characteristics to create sulcal and gyral regions. Common indices for evaluating brain gyrification are the gyrification index (GI) and sulcal depth.^24^ The clinical relevance of these indices has been suggested in studies of healthy and delayed brain development.^24,25,26,27,28,29,30^ However, to our knowledge, the association of SES with brain gyrification has rarely been explored,^3,15,31^ with less research for the fetal brain.

To characterize 3-dimensional fetal brain morphology, 3 cortical features (ie, lobe volume, local GI [LGI], and sulcal depth) were measured in the frontal, parietal, temporal, and occipital lobes (eFigure 4 in the Supplement).^30,32,33,34^ Volumes of the 4 lobes of WM were calculated as the multiplication of the number of brain voxels by the voxel resolution from the magnetic resonance images.^29^ To calculate LGI and sulcal depth, convex hull surface of the connected brain regions was first generated.^35^ The LGI at a given vertex on the surface was computed as the ratio between the area of a circular region of the vertex on the surface and the corresponding area on the convex hull for the vertex.^26,36,37^ The sulcal depth was characterized by the distance from each vertex on the brain surface to the nearest point on a convex hull for each brain hemisphere.^38^ The LGI and sulcal depth were calculated on the inner surface of the CGM (ie, the gray and white junction).^25,30,32,39^

### Maternal Distress

Maternal distress measures were completed by each woman on the day of the MRI. These measures included the Spielberger State Anxiety Inventory (SSAI), Spielberger Trait Anxiety Inventory (STAI), Perceived Stress Scale (PSS), and Edinburgh Postnatal Depression Scale (EPDS).^40^ Of the study group, only 84 participants (58.3%) completed these questionnaires given that they were included in our design from 2015 onward.

### Statistical Analysis

Various univariate analyses were performed to explore the demographic data and SES measures. The Kolmogorov-Smirnov test was used to test the normality of the continuous variables, including gestational age (GA); maternal age; child’s birth weight, length, and head circumference; parental SES scores; and maternal distress measures, and the results suggested that all these variables were not normally distributed. The fetal and maternal demographic characteristics were therefore compared between families with low SES and those with high SES using Wilcoxon-Mann-Whitney tests for GA, maternal age; *z* scores of child’s birth weight, length, and head circumference; and maternal distress measures and using χ^2^ tests for fetal sex, number of scans, maternal parity, and maternal race/ethnicity. The high SES group included those families whose maternal or paternal SES score was higher than the median SES score (51). The association between maternal education and occupation levels and paternal education and occupation levels were assessed using χ^2^ tests. The maternal and paternal SES scores were compared using Wilcoxon-Mann-Whitney test. The correlations among SES measures (ie, education and occupation levels and SES score) were reported by Spearman correlation coefficient (*ρ*). The somatic size (ie, birth height, length, and head circumference) by SES (low vs high), adjusted for GA at birth and fetal sex, was further analyzed using linear regression. Of 40 participants who had 2 MRIs, the slopes to measure brain growth rate through the difference of brain volumes and cortical features between the first and second MRIs were calculated, and statistical comparisons of the slopes between low and high SES using Wilcoxon-Mann-Whitney tests were conducted.

The associations between SES measures and fetal brain tissue volumes and cortical features were analyzed using linear mixed-effects models.^41,42^ More specifically, in each linear mixed-effects model, 1 of the following dependent variables was considered: brain tissue volume for the 5 brain tissues; whole brain volume; or lobe volume, LGI, or sulcal depth for the 4 lobes. The primary independent variable was education, occupation, or SES score as a continuous or dichotomized (ie, low SES, 0; high SES, 1) variable, and all linear mixed-effects models were adjusted for GA at MRI date (in weeks) and fetal sex (ie, female, 0; male, 1). Random association using Cholesky parameterization was included to control intrasubject correlation owing to the potential for more than 1 scan for each woman.^41,43^

We further incorporated maternal distress (ie, low distress, 0; high distress, 1) into the linear mixed-effects models to investigate these associations after adjusting for maternal distress. High distress was defined as a participant in an MRI visit with 1 of the 4 distress measures greater than the corresponding threshold (ie, SSAI, 40; STAI, 40; PSS, 15; EPDS, 10).^40^ The associations between maternal distress measures and parental education, occupation levels, and SES score were determined using linear mixed-effects models.

The critical value for significance was set as .05.^44^ The *q* values calculated by the Benjamini-Hochberg method for 5 brain tissue types or 4 brain lobes were also reported to reflect significant parameters under multiple comparisons. All analyses performed in this study were conducted from June through November 2020 using Matlab statistical software version R2019a (MathWorks),^41^ and all hypothesis tests were 2-sided.

## Results

### Baseline Demographic Characteristics and SES

A diagram illustrating participant recruitment is shown in eFigure 5 in the Supplement. Of 286 MRI scans taken, 29 MRI (10.1%) scans with excessive motion and 73 MRI scans (25.5%) with failed brain surface reconstruction for cortical folding measures were excluded. The final data set consisted of 144 pregnant women (median [interquartile range] age, 32.5 [27.0-36.1] years); 75 fetuses (52.1%) were male, and 69 fetuses (47.9%) were female. A total of 184 fetal brain MRI scans were acquired (Table 1). Among 144 study participants, 40 fetuses (23 male fetuses and 17 female fetuses) were scanned at 2 points during pregnancy, while all other fetuses (52 male fetuses and 52 female fetuses) were scanned once. The median (range) GA at MRI of the 184 scans was 33.6 (24.0-39.4) weeks. Of 40 study participants who had 2 MRI scans, the median (range) GA was 28.3 (25.0-36.1) weeks for the first MRI scan and 36.6 (31.3-39.1) for the second MRI scan. Table 2 shows that about 40% of parents had graduate professional training (59 mothers [41.0%] and 56 fathers [38.9%]), while less than 25% had high school or partial high school education (23 mothers [16.0%] and 35 fathers [24.3%]). Approximately 60% of parents had a major business or professional occupation (85 mothers [59.0%] and 87 fathers [60.4%]). Among all participants, 27 mothers were unemployed or homemakers (18.8%), vs 18 fathers who were unemployed or homemakers (12.5%). No significant differences between the distributions of maternal and paternal education or occupation levels were found (Table 2). The median (range) maternal and paternal SES scores were both 51 (11-55), and the maternal and paternal SES scores were not significantly different (rank sum test statistic, 20 841; *P* = .96). All SES measures were significantly correlated (eTable 1 in the Supplement). In addition, birth weight, length, and head circumference, adjusting for GA at birth and fetal sex, did not differ significantly between the low and high SES groups based on linear regression. The number of MRI scans obtained for each GA range is shown in eTable 2 in the Supplement.

**Table 1.** Demographic Characteristics of Mothers

Characteristic	No (%)			*P* value
	Total	Low SES group	High SES group	
Total participants	144	72 (50.0)	72 (50.0)	NA
Sex of fetus				
Female	69 (47.9)	35 (48.6)	34 (47.2)	.87
Male	75 (52.1)	37 (51.4)	38 (52.8)	
No. of scans				
1	104 (72.2)	55 (76.4)	49 (68.1)	.31
2	40 (27.8)	17 (23.6)	23 (31.9)	
MRI scans	184	89 (48.4)	95 (51.6)	NA
Time point 1	85 (46.2)	44 (49.4)	41 (43.2)	.39
Time point 2	99 (53.8)	45 (50.6)	54 (56.8)	
GA, median (range), wk				
At MRI overall	33.6 (29.3 to 36.1)	33.0 (29.3 to 35.9)	33.7 (29.3 to 36.4)	.50
At MRI time point 1	29.1 (27.7 to 31.1)	29.2 (27.6 to 31.1)	29.1 (27.6 to 30.9)	.76
At MRI time point 2	35.9 (34.7 to 37.0)	35.7 (34.6 to 37.0)	36.1 (34.7 to 37.0)	.76
At birth^a^	39.6 (38.7 to 40.3)	39.3 (38.4 to 40.0)	39.7 (38.9-40.5)	.04
Maternal age, median (IQR), y^b^	32.5 (27.0 to 36.1)	26.6 (22.2 to 32.5)	34.9 (32.4 to 38.2)	<.001
Maternal parity ^c^				
Primiparous	67 (46.5)	27 (37.5)	40 (55.6)	.01
Multiparous	68 (47.2)	42 (58.3)	26 (36.1)	
Child characteristics at birth, median (IQR), *z* score				
Weight, kg^d^	0.07 (−0.60 to 0.63)	−0.12 (−0.68 to 0.57)	0.28 (−0.42 to 0.76)	.10
Length, cm^e^	0.04 (−0.75 to 0.60)	−0.21 (−0.99 to 0.28)	0.12 (−0.37 to 0.71)	.15
Head circumference, cm^f^	−0.26 (−0.59 to 0.75)	−0.26 (−0.93 to 0.42)	0.08 (−0.59 to 0.75)	.15
Mothers’ race/ethnicity				
White	68 (47.2)	16 (22.2)	52 (72.2)	<.001
Black	45 (31.3)	40 (55.6)	5 (6.9)	
Hispanic or Latino	16 (11.1)	7 (9.7)	9 (12.5)	
Asian or Pacific Islander	6 (4.2)	3 (4.2)	3 (4.2)	
Other or unknown	9 (6.3)	6 (8.3)	3 (4.2)	

Abbreviations: GA, gestational age; IQR, interquartile range; MRI, magnetic resonance imaging; NA, not applicable; SES, socioeconomic status.

^a^ Based on 134 participants.

^b^ Based on 134 participants.

^c^ Based on 135 participants.

^d^ Based on 122 participants.

^e^ Based on 78 participants.

^f^ Based on 78 participants.

**Table 2.** Participant Education and Occupation Levels

Level	Education	No (%)		Occupation	No (%)	
		Mothers	Fathers		Mothers	Fathers
1	Partial high school	4 (2.8)	9 (6.3)	Homemaker, unemployed	27 (18.8)	18 (12.5)
2	High school graduate	19 (13.2)	26 (18.1)	Unskilled laborer, machine operative, semiskilled operator	9 (6.3)	15 (10.4)
3	Partial college (at least 1 y)	23 (16.0)	23 (16.0)	Skilled craftsman, clerical, sales	8 (5.6)	14 (9.7)
4	College or university graduate	39 (27.1)	30 (20.8)	Medium business, minor professional, technical	15 (10.4)	10 (6.9)
5	Graduate professional training	59 (41.0)	56 (38.9)	Major business, professional	85 (59.0)	87 (60.4)
χ^2^^a^		4.26			5.96	
*P* value		.37			.20	

^a^ χ^2^ tests were used to detect the association between maternal and paternal education and occupation levels.

Fetal CGM volumetric growth rate was higher in the low SES group, while WM volumetric growth rate was higher in the high SES group (eTable 3 in the Supplement). No significant differences in LGI or sulcal depth growth rates were found between low and high SES groups (eTable 3 in the Supplement).

None of the maternal distress measures were different between low and high SES groups (eTable 4 in the Supplement). No significant associations between maternal distress measures and parental education or occupation levels or SES score were found (eTable 5 in the Supplement).

### Fetal Brain Tissue Volumes

We found that higher parental education, occupation, and SES score were associated with significantly increased fetal brain WM, DGM, brainstem, and cerebellar volumes (Table 3; eFigure 6 in the Supplement). Specifically, higher maternal and paternal education levels were associated with significantly increased volumes of the WM (mothers: β, 2.86; 95% CI, 1.26-4.45; *P* = .001; fathers: β, 2.39; 95% CI, 0.97-3.81; *P* = .001), DGM (mothers: β, 0.16; 95% CI, 0.002-0.32; *P* = .048; fathers: β, 0.16; 95% CI, 0.02-0.31; *P* = .02), and brainstem (mothers: β, 0.06; 95% CI, 0.02-0.10; *P* = .01; fathers: β, 0.04; 95% CI, 0.004-0.08; *P* = .03). In addition, higher maternal occupation status was associated with significantly increased volumes in the WM (β, 2.07; 95% CI, 0.88-3.26; *P* = .001), brainstem (β, 0.03; 95% CI, 0.001-0.07; *P* = .04), and cerebellum (β, 0.17; 95% CI, 0.04-0.29; *P* = .01), while higher paternal occupation status was associated with significantly increased WM volume (β, 1.98; 95% CI, 0.71-3.25; *P* < .01). Higher maternal SES score was associated with significantly increased volume of the WM (β, 0.23; 95% CI, 0.11-0.36; *P* < .001), brainstem (β, 0.004; 95% CI, 0.001-0.01; *P* = 0.02), and cerebellum (β, 0.02; 95% CI, 0.01-0.03; *P* = .004), and higher paternal SES score was associated with significantly increased volume in the WM (β, 0.21; 95% CI, 0.09-0.34; *P* = .001) and brainstem (β, 0.004; 95% CI, 0.0001-0.007; *P*=0.04).

**Table 3.** Associations Between Parental Socioeconomic Measures and Fetal Brain Volumes^a^

	Education level		Occupation level		SES score	
	β (95% CI)	*P* value	β (95% CI)	*P* value	β (95% CI)	*P* value
**Mothers**						
CGM	−1.41 (−2.41 to −0.41)	.01^b^	−0.90 (−1.65 to −0.16)	.02^b^	−0.11 (−0.18 to −0.03)	.01^b^
WM	2.86 (1.26 to 4.45)	.001^b^	2.07 (0.88 to 3.26)	.001^b^	0.23 (0.11 to 0.36)	<.001^b^
Cerebellum	0.26 (0.09 to 0.42)	.003^b^	0.17 (0.04 to 0.29)	.01^b^	0.02 (0.01 to 0.03)	.004^b^
DGM	0.16 (0.002 to 0.32)	.048	0.09 (−0.03 to 0.21)	.12	0.01 (−0.001 to 0.02)	.08
Brainstem	0.06 (0.02 to 0.10)	.01^b^	0.03 (0.001 to 0.07)	.04	0.004 (0.001 to 0.01)	.02^b^
Whole brain	1.92 (−0.20 to 4.03)	.08	1.46 (−0.11 to 3.03)	.07	0.16 (−0.004 to 0.33)	.06
**Fathers**						
CGM	−0.95 (−1.84 to −0.06)	.04	−1.08 (−1.87 to −0.29)	.01^b^	−0.10 (−0.18 to −0.03)	.01^b^
WM	2.39 (0.97 to 3.81)	.001^b^	1.98 (0.71 to 3.25)	.002^b^	0.21 (0.09 to 0.34)	.001^b^
Cerebellum	0.13 (−0.02 to 0.28)	.09	0.08 (−0.06 to 0.21)	.25	0.01 (−0.004 to 0.02)	.16
DGM	0.16 (0.02 to 0.31)	.02	0.11 (−0.02 to 0.24)	.10	0.01 (−0.0001 to 0.03)	.05
Brainstem	0.04 (0.004 to 0.08)	.03	0.03 (−0.002 to 0.07)	.07	0.004 (0.0001 to 0.007)	.04
Whole brain	1.78 (−0.09 to 3.64)	.06	1.12 (−0.56 to 2.79)	.19	0.13 (−0.03 to 0.30)	.12

Abbreviations: CGM, cortical gray matter; DGM, deep gray matter; GA, gestational age; SES, socioeconomic status; WM, white matter.

^a^ Adjusted for GA at magnetic resonance imaging scan (wk) and fetal sex (ie, female: 0; male: 1). Brain volume measured in cm^3^.

^b^ *q* < 0.05.

Higher level of parental education was associated with significantly decreased CGM volume (mothers: β, −1.41; 95% CI, −2.41 to −0.41; *P* = .01; fathers: β, −0.95; 95% CI, −1.84 to −0.06; *P* = 0.04), as were higher occupation level (mothers: β, −0.90; 95% CI, −1.65 to −0.16; *P* = .02; fathers: β, −1.08; 95% CI, −1.87 to −0.29; *P* = .01) and SES score (mothers: β, −0.11; 95% CI, −0.18 to −0.03; *P* = .01; fathers: β, −0.10; 95% CI, −0.18 to −0.03; *P* = .01). In the high SES group, compared with the low SES group, the CGM volume was lower (68.1 cm^3^ vs 70.8 cm^3^) while the volume was higher in the WM (112.7 cm^3^ vs 106.8 cm^3^), cerebellum (10.9 cm^3^ vs 10.5 cm^3^), and brainstem (4.65 cm^3^ vs 4.53 cm^3^) (eTable 6 and eFigure 7 in the Supplement).

### Fetal Cortical Folding

Regional cortical features were further calculated for 4 lobes. Higher maternal SES score was associated with increased lobe volume: frontal lobe (β, 0.07; 95% CI, 0.02-0.13; *P* = .01), parietal lobe (β, 0.07; 95% CI, 0.03-0.11; *P* < .001), temporal lobe (β, 0.04; 95% CI, 0.02-0.07; *P* < .001), and occipital lobe (β, 0.02; 95% CI, 0.01-0.04; *P* = .03). Higher maternal education level (frontal lobe: β, 0.91; 95% CI, 0.22-1.59; *P* = .01; parietal lobe: β, 0.90; 95% CI, 0.43-1.37; *P* < .001; temporal lobe: β, 0.54; 95% CI, 0.27-0.82; *P* < .001; occipital lobe: β, 0.23; 95% CI, 0.02-0.45; *P* = .03) and occupation level (frontal lobe: β, 0.67; 95% CI, 0.16-1.17; P=0.01; parietal lobe: β, 0.62; 95% CI, 0.27-0.97; *P* = .001; temporal lobe: β, 0.38; 95% CI, 0.17-0.59; *P* < .001; occipital lobe: β, 0.17; 95% CI, 0.01-0.33; *P* = .04) were also associated with larger lobe volumes (Table 4; eFigure 8 in the Supplement).

**Table 4.** Associations Between Maternal Socioeconomic Measures and Brain Lobe Volumes and Cortical Features^a^

	Lobe volume, cm^3^		LGI × 10^−3^		Sulcal depth × 10^−3^ mm	
	β (95% CI)	*P* value	β (95% CI)	*P* value	β (95% CI)	*P* value
**Education level**						
Frontal lobe	0.91 (0.22 to 1.59)	.01^b^	−13 (−23 to −3)	.01^b^	−25 (−64 to 15)	.22
Parietal lobe	0.90 (0.43 to 1.37)	<.001^b^	−29 (−45 to −14)	<.001^b^	−116 (−171 to −62)	<.001^b^
Temporal lobe	0.54 (0.27 to 0.82)	<.001^b^	−18 (−30 to −6)	.005^b^	−62 (−105 to −18)	.01^b^
Occipital lobe	0.23 (0.02 to 0.45)	.03^b^	−28 (−42 to −15)	<.001^b^	−94 (−136 to −53)	<.001^b^
**Occupation level**						
Frontal lobe	0.67 (0.16 to 1.17)	.01^b^	−10 (−17 to −3)	.01^b^	−22 (−52 to 7)	.13
Parietal lobe	0.62 (0.27 to 0.97)	.001^b^	−22 (−33 to −10)	<.001^b^	−85 (−125 to −44)	<.001^b^
Temporal lobe	0.38 (0.17 to 0.59)	<.001^b^	−13 (−22 to −4)	.005^b^	−48 (−81 to −16)	.003^b^
Occipital lobe	0.17 (0.01 to 0.33)	.04^b^	−18 (−28 to −8)	.001^b^	−64 (−95 to −33)	<.001^b^
**SES score**						
Frontal lobe	0.07 (0.02 to 0.13)	.01^b^	−1.1 (−1.9 to −0.3)	.01^b^	−2.4 (−5.5 to 0.7)	.14
Parietal lobe	0.07 (0.03 to 0.11)	<.001^b^	−2.4 (−3.6 to −1.2)	<.001^b^	−9.5 (−13.8 to −5.3)	<.001^b^
Temporal lobe	0.04 (0.02 to 0.07)	<.001^b^	−1.5 (−2.4 to −0.5)	.003^b^	−5.3 (−8.7 to −1.9)	.002^b^
Occipital lobe	0.02 (0.002 to 0.04)	.03^b^	−2.1 (−3.2 to −1.0)	<.001^b^	−7.3 (−10.6 to −4.1)	<.001^b^

Abbreviations: GA, gestational age; LGI, local gyrification index; SES, socioeconomic status.

^a^ Adjusted for GA at magnetic resonance imaging scan (wk) and fetal sex (ie, female: 0; male: 1).

^b^ *q* < 0.05.

Higher maternal SES score was associated with significantly decreased LGI (for example, for the frontal lobe: β, −1.1; 95% CI, −1.9 to −0.3) and sulcal depth (for example, for the parietal lobe: β, −9.5; 95% CI, −13.8 to −5.3; *P* < .001), except for sulcal depth in the frontal lobe. Higher maternal education level was also associated with significantly decreased LGI (for example, for the frontal lobe: β, −13; 95% CI, −23 to −3; *P* = .01) and sulcal depth (for example, for the parietal lobe: β, −116; 95% CI, −171 to −62; *P* < .001), as was maternal occupation level (for example, LGI for the frontal lobe: β, −10; 95% CI, −17 to −3; *P* = .01 and sulcal depth for the parietal lobe: β, −85; 95% CI, −125 to −44; *P* < .001), except for sulcal depth in the frontal lobe.

Similarly, higher paternal SES score was associated with significantly increased lobe volumes, except for the occipital lobe (frontal lobe: β, 0.06; 95% CI, 0.01 to 0.11; *P* = .03; parietal lobe: β, 0.06; 95% CI, 0.03 to 0.10; *P* = .001; temporal lobe: β, 0.04; 95% CI, 0.02 to 0.07; *P* < .001). Higher paternal education (frontal lobe: β, 0.71; 95% CI, 0.11-1.32; *P* = .02; parietal lobe: β, 0.69; 95% CI, 0.27-1.11; *P* = .002; temporal lobe: β, 0.45; 95% CI, 0.20-0.70; *P* < .001; occipital lobe: β, 0.21; 95% CI, 0.02-0.40; *P* = .03) and occupation level (frontal lobe: β, 0.55; 95% CI, 0.01-1.10; *P* = .046; parietal lobe: β, 0.62; 95% CI, 0.25-1.00; *P* = .001; temporal lobe: β, 0.44; 95% CI, 0.22-0.66; *P* < .001) were also associated with significantly larger lobe volume, except for paternal occupation level and the occipital lobe (Table 5; eFigure 8 in the Supplement).

**Table 5.** Associations Between Paternal Socioeconomic Measures and Brain Lobe Volumes and Cortical Features^a^

	Lobe volume, cm^3^		LGI, × 10^−3^		Sulcal depth × 10^−3^ mm	
	β (95% CI)	*P* value	β (95% CI)	*P* value	β (95% CI)	*P* value
**Education level**						
Frontal lobe	0.71 (0.11 to 1.32)	.02^b^	−9 (−18 to −0.1)	.048^b^	−15 (−50 to 20)	.40
Parietal lobe	0.69 (0.27 to 1.11)	.002^b^	−24 (−37 to −10)	.001^b^	−90 (−139 to −41)	<.001^b^
Temporal lobe	0.45 (0.20 to 0.70)	<.001^b^	−12 (−23 to −1)	.03^b^	−53 (−91 to −14)	.01^b^
Occipital lobe	0.21 (0.02 to 0.40)	.03^b^	−22 (−34 to −10)	<.001^b^	−78 (−115 to −41)	<.001^b^
**Occupation level**						
Frontal lobe	0.55 (0.01 to 1.10)	.046	−8 (−16 to −0.3)	.04^b^	−16 (−48 to 15)	.30
Parietal lobe	0.62 (0.25 to 1.00)	.001^b^	−21 (−33 to −9)	.001^b^	−85 (−128 to −42)	<.001^b^
Temporal lobe	0.44 (0.22 to 0.66)	<.001^b^	−14 (−23 to −4)	.01^b^	−51 (−85 to −17)	.004^b^
Occipital lobe	0.14 (−0.04 to 0.31)	.12	−18 (−29 to −7)	.001^b^	−65 (−98 to −31)	<.001^b^
**SES score**						
Frontal lobe	0.06 (0.01 to 0.11)	.03^b^	−0.8 (−1.6 to −0.1)	.03^b^	−1.6 (−4.7 to 1.5)	.31
Parietal lobe	0.06 (0.03 to 0.10)	.001^b^	−2.2 (−3.4 to −1.0)	<.001^b^	−8.7 (−13.0 to −4.4)	<.001^b^
Temporal lobe	0.04 (0.02 to 0.07)	<.001^b^	−1.3 (−2.3 to −0.4)	.01^b^	−5.1 (−8.5 to −1.8)	.003^b^
Occipital lobe	0.02 (−0.001 to 0.03)	.07	−1.9 (−3.0 to −0.9)	<.001^b^	−6.9 (−10.2 to −3.6)	<.001^b^

Abbreviations: GA, gestational age; LGI, Local gyrification index; SES, socioeconomic status.

^a^ Adjusted for GA at magnetic resonance imaging scan (wk) and fetal sex (ie, female: 0; male: 1).

^b^ *q* < 0.05.

In addition, higher paternal SES score was associated with significantly decreased LGI (for example, for the frontal lobe: β, −0.8; 95% CI, −1.6 to −0.1; *P* = .03) and sulcal depth (for example, for the parietal lobe: β, −8.7; 95% CI, −13.0 to −4.4; *P* < .001), except for sulcal depth in the frontal lobe. Higher paternal education was also associated with significantly decreased LGI (for example, for the frontal lobe: β, −9; 95% CI, −18 to −0.1; *P* = .048) and sulcal depth (for example, for the parietal lobe: β, −90; 95% CI, −139 to −41; *P* < .001), as was higher occupation level, (for example, LGI for the frontal lobe: β, −8; 95% CI, −16 to −0.3; *P* = .04 and sulcal depth for the parietal lobe: β, −85; 95% CI, −128 to −42; *P* < .001), except for sulcal depth in the frontal lobe.

In the high SES group, compared with the low SES group, LGI was decreased in the 4 brain lobes (frontal lobe: 1.22 vs 1.25; parietal lobe: 1.34 vs 1.40; temporal lobe: 1.34 vs 1.37; occipital lobe: 1.27 vs 1.32), and sulcal depth was decreased in the parietal lobe (2.00 mm vs 2.25 mm), temporal lobe (1.85 mm vs 1.97 mm), and occipital lobe (1.17 mm vs 1.32 mm) (eTable 6 and eFigure 9 in the Supplement).

All β coefficients of GA at MRI were positive and significant (eTable 7, eTable 8, eTable 9 in the Supplement). Male fetuses had higher CGM and brainstem volume and total brain volume (eTable 10 in the Supplement), while there were no differences in LGI or sulcal depth by sex (eTable 11 and eTable 12 in the Supplement).

After further adjusting for maternal distress, we found that higher parental education level, occupation level, and SES score were associated with increased WM and total brain volume (eTable 13 in the Supplement). After the adjustment, increased maternal education was associated with decreased LGI and sulcal depth in the parietal lobe and no paternal SES measures were associated with LGI or sulcal depth (eTable 14 and eTable 15 in the Supplement).

## Discussion

This cohort study found that parental SES was associated with altered tissue volume in several brain regions and altered cortical features among fetuses. The study used advanced in vivo fetal 3-dimensional volumetric MRI to investigate the association between SES and in utero fetal brain development during the latter half of gestation. Quantitative fetal MRI techniques afforded us the unique opportunity to noninvasively probe in utero fetal brain developmental trajectories. To the best of our knowledge, this study is the first to examine in utero regional and tissue-specific brain growth and emerging cerebral cortical folding in association with socioeconomic indicators in a large normative population sample of healthy fetuses from healthy pregnancies. We found that SES measures (ie, education level, occupation level, and SES score) were mutually correlated. Fetal CGM volume was decreased while WM, cerebellar, and brainstem volumes were increased in the high SES group. For our cortical measures, LGI and sulcal depth were decreased in the high SES group. We also found that higher maternal and paternal education levels were associated with increased DGM and brainstem volumes, while higher maternal occupation level was associated with increased cerebellar and brainstem volumes. Conversely, higher SES measures were associated with decreased LGI and sulcal depth in all lobes, except for sulcal depth in the frontal lobe. These findings suggest that parental education level and employment status are associated with in vivo human fetal brain development and may provide evidence for altered prenatal programming.

We found that higher parental education and occupation levels were associated with increased volumes of the developing fetal brain WM, DGM, cerebellum, and brainstem during pregnancy. The association of SES with the volumes of specific brain lobes or regions has been previously investigated among infants and older children, and the results of these studies^2,9,10,11,45^ are consistent with our findings in the prenatal period. A review study^46^ found that several potential proximal factors, such as stress, parenting practices, prenatal care, and nutrition, may be associated with SES measures and brain structure and function. Although the mechanisms underlying our in utero observations are undoubtedly complicated and poorly understood, we postulate that increased psychosocial stress in populations with lower SES may be associated with WM development in the fetal brain. Four studies^47,48,49,50^ have found that lower family income is associated with higher stress prevalence, and it is well known that poverty is associated with a variety of negative life experiences and higher levels of stress.^51,52,53,54^ Collectively, these stressful life events may be significant mediators associated with income-to-need ratio and childhood brain development.^11^ These associations may also be explained by additional psychological stressors.^51,52^ Maternal education has also been found to be associated with brain development and verbal scale IQ of offspring by predominantly genetic associations, in addition to shared environmental associations.^55^ These risk factors may play a role in the association between parental SES and altered fetal brain volumes.^11^ Of note, these risk factors may be further elucidated using mediation analysis for measures of SES, candidate proximal mediators, and analyses of the brain measures.^46,56^

We found that higher SES was associated with decreased CGM volume. Although the mechanisms underlying these associations are unclear, we postulate that lower SES is associated with increased CGM volume secondary to an inflammatory response associated with increased stress levels, representing a compensatory outcome in the early stage of mental illness.^57,58,59,60^ Studies^61,62,63^ have found that stress is associated with increased inflammatory responses and altered cytokine production during gestation. However, it should be noted that Wu et al^40^ reported in 2020 that increased maternal psychological distress during pregnancy in a cohort of women with high levels of resources and education was not associated with impaired fetal brain tissue growth or cerebellar or brainstem volume. Rather, increased maternal stress was associated with decreased fetal hippocampal volume, suggesting regional vulnerability of the hippocampal structure. These data suggest that the underlying mechanisms and mediators associated with stress in the setting of SES-associated disparities and altered fetal neurodevelopment may be different. Results in the limited literature suggest that lower SES is associated with decreased volumes of CGM; however, it is worthwhile to note that these findings have generally been in the context of studies designed to address targeted brain regions in children and adolescents.^2,8,9,11^ In our analyses, SES measures were not associated with variations in total brain volumes. However, higher SES measures were associated with decreased regional CGM volume and increased WM volume. These findings are consistent with the results of studies from 2012^10,14^ and 2010^13^ among older children and young adolescents. Future prospective longitudinal studies may be needed to address these intriguing and complex knowledge gaps.

We reported that higher SES measures were associated with increased LGI and sulcal depth on the cortical surface in the parietal, temporal, and occipital lobes. This accelerated brain gyrification in lower SES populations may be in part associated with parental stress. Converging evidence points to increased cerebral cortical folding in fetuses,^40^ children, and adults^64,65,66,67,68^ exposed to prenatal stress and other neuropsychiatric disorders, including autism and schizophrenia. Studies have reported increased GI in the frontal and temporal lobes in adults with schizophrenia^64,65,66,67^ and in the frontal lobe of children with autism.^68^ These findings suggest that parental psychological distress in the setting of lower SES across the lifespan may play a role in the complex cerebral cortical folding process, and this risk may have a fetal onset.

Several studies^7,8,10,11,13,16^ have reported an association between SES and brain volumes among infants and adolescents or reported the association of SES with neurodevelopmental outcomes after birth.^69,70,71,72,73^ However, to the best of our knowledge, only 1 study^10^ investigated the association between SES and gyrification, finding positive associations between GI and SES in the anterior frontal regions of the left hemisphere. While that conclusion may seem to contradict our findings, several distinguishing features should be noted. First, Jednorog et al’s study^10^ consisted of a small cohort of 23 children (aged 8-10 years), while our study included a large cohort of 184 fetuses. Second, that study’s GI calculations were based on the cerebral pial surface, vs the gray and white junction used in our study. This junction is known to be more reliably identified than the pial surface,^39^ and most published GI calculations have been based on the gray and white junction, including studies among fetuses,^30,32^ preterm infants,^25,28,30,33^ term-born neonates,^34^ children,^39,74^ and adults.^35,75^ Ongoing studies may be needed to disentangle the complex role and interplay of prenatal parental SES from postnatal SES associations.

### Limitations

This study has several limitations. First, family income data were not available in our cohort, and therefore we could not examine associations between income and fetal brain development. However, we used occupation level to represent financial standing, which was similar to the strategies used in other studies.^2,10^ In addition, more detailed assessments of the associations between poverty and indicators associated with physiological and psychological outcomes may be needed to further elucidate mechanisms associated with risk. These indicators may include nutrition and food security, familial psychopathology, and genetic factors, such as parental somatic size. Furthermore, 113 of the women (78.5%) in our study group were White or Black. Race and ethnicity, which are known to be associated with differences in adult brain structure,^76,77^ should be further explored in future studies to best reflect the diversity of the general population. In addition, we found no difference in distress measures between the group with low SES and that with high SES, and we found no differences in the associations between SES measures and brain volume or cortical features after adjusting for maternal stress, anxiety, and depression. However, measures of maternal psychological distress were available for approximately 60% of the study group, given that we began capturing maternal distress measures in 2015. These results suggest further investigation of potential mediators in these associations may be needed to explore the underlying mechanisms. Additionally, the long-term neurodevelopmental associations of these in vivo fetal brain alterations as measured by prenatal quantitative MRI are unknown and currently under investigation.

## Conclusions

This cohort study found an association between parental socioeconomic status and altered in vivo fetal neurodevelopment. Understanding how parental SES variations may be associated with neural, social-emotional, and cognitive functioning in humans has major implications for basic scientific questions and public policy initiatives. Greater insights into the neural underpinnings associated with SES, specifically education and poverty, may aid in the design and implementation of intervention programs addressing SES-associated disparities in cognitive and health outcomes. To the best of our knowledge, this is the first longitudinal study to examine the associations of parental education and occupation with fetal neurodevelopment. These findings may increase our understanding of the neural mechanisms associated with socioeconomic disparities in fetal brain development. We believe that these findings of altered volumetric growth may inform preventive educational strategies and targeted early interventions for the low SES population, which faces a multitude of physical and psychosocial stressors. Ongoing studies are needed and currently underway to determine the long-term association of SES with neurodevelopmental outcomes in infancy and early childhood.

## Notes

Supplement. **eTable 1.** Spearman Correlation Coefficients Among Socioeconomic Status Measures **eTable 2.** Magnetic Resonance Imaging Scans Obtained for Each Gestational Age Range **eTable 3.** Brain Growth Rate vs Gestational Age Between First and Second Scans **eTable 4.** Distress Measures **eTable 5.** Associations Between Maternal Distress Measures and Parental Socioeconomic Status Measures **eTable 6.** Associations Between Fetal Brain Volume and Cortical Features and Parental Socioeconomic Status Measures **eTable 7.** β Coefficients of Gestational Age for Associations Between Fetal Brain Volume and Parental Socioeconomic Status Measures **eTable 8.** β Coefficients of Gestational Age for Associations Between Brain Lobe Volume and Cortical Features and Maternal Socioeconomic Measures **eTable 9.** β Coefficients of Gestational Age for Associations Between Brain Lobe Volume and Cortical Features and Paternal Socioeconomic Measures **eTable 10.** β Coefficients of Fetal Sex for Associations Between Fetal Brain Volume and Parental Socioeconomic Status Measures **eTable 11.** β Coefficients of Fetal Sex for Associations Between Brain Lobe Volume and Cortical Features and Maternal Socioeconomic Status Measures **eTable 12.** β Coefficients of Fetal Sex for Associations Between Brain Lobe Volume and Cortical Features and Paternal Socioeconomic Status Measures **eTable 13.** Associations Between Fetal Brain Volumes and Parental Socioeconomic Status Measures **eTable 14.** Associations Between Brain Lobe Volumes and Cortical Features and Maternal Socioeconomic Status Measures **eTable 15.** Associations Between Brain Lobe Volume and Cortical Features and Paternal Socioeconomic Status Measures **eFigure 1.** Fetal Brain Magnetic Resonance Axial Plane Images and Reconstructed White Matter Surface at Different Gestational Ages **eFigure 2.** Brain Tissue Segmentation **eFigure 3.** Brain Parcellation **eFigure 4.** Cortical Folding **eFigure 5.** Subject Recruitment Flowchart **eFigure 6.** Brain Tissue Volume vs Socioeconomic Status Score **eFigure 7.** Brain Tissue Volume vs Gestational Age **eFigure 8.** Cerebral Cortical Features vs Socioeconomic Status Score **eFigure 9.** Cerebral Cortical Features vs Gestational Age
