# Supplementary material for: Association Between Socioeconomic Status and In Utero Fetal Brain Development
Source: JAMA Netw Open. 2021 Mar 29;4(3):e213526. doi: 10.1001/jamanetworkopen.2021.3526 (PMC8008281; doi:10.1001/jamanetworkopen.2021.3526)
Supplement: Supplement. — eTable 1. Spearman Correlation Coefficients Among Socioeconomic Status Measures eTable 2. Magnetic Resonance Imaging Scans Obtained for Each Gestational Age Range eTable 3. Brain Growth Rate vs Gestational Age Between First and Second Scans eTable 4. Distress Measures eTable 5. Associations Between Maternal Distress Measures and Parental Socioeconomic Status Measures eTable 6. Associations Between Fetal Brain Volume and Cortical Features and Parental Socioeconomic Status Measures eTable 7. β Coefficients of Gestational Age for Associations Between Fetal Brain Volume and Parental Socioeconomic Status Measures eTable 8. β Coefficients of Gestational Age for Associations Between Brain Lobe Volume and Cortical Features and Maternal Socioeconomic Measures eTable 9. β Coefficients of Gestational Age for Associations Between Brain Lobe Volume and Cortical Features and Paternal Socioeconomic Measures eTable 10. β Coefficients of Fetal Sex for Associations Between Fetal Brain Volume and Parental Socioeconomic Status Measures eTable 11. β Coefficients of Fetal Sex for Associations Between Brain Lobe Volume and Cortical Features and Maternal Socioeconomic Status Measures eTable 12. β Coefficients of Fetal Sex for Associations Between Brain Lobe Volume and Cortical Features and Paternal Socioeconomic Status Measures eTable 13. Associations Between Fetal Brain Volumes and Parental Socioeconomic Status Measures eTable 14. Associations Between Brain Lobe Volumes and Cortical Features and Maternal Socioeconomic Status Measures eTable 15. Associations Between Brain Lobe Volume and Cortical Features and Paternal Socioeconomic Status Measures eFigure 1. Fetal Brain Magnetic Resonance Axial Plane Images and Reconstructed White Matter Surface at Different Gestational Ages eFigure 2. Brain Tissue Segmentation eFigure 3. Brain Parcellation eFigure 4. Cortical Folding eFigure 5. Subject Recruitment Flowchart eFigure 6. Brain Tissue Volume vs Socioeconomic Status Score eFigure 7. Brain Tiss [file jamanetwopen-e213526-s001.pdf]

Supplemental Online Content

Lu YC, Kapse K, Andersen N, et al. Association between socioeconomic status and in utero fetal brain development. *JAMA Netw Open*. 2021;4(3):e213526.
doi:10.1001/jamanetworkopen.2021.3526

eTable 1. Spearman Correlation Coefficients Among Socioeconomic Status Measures

eTable 2. Magnetic Resonance Imaging Scans Obtained for Each Gestational Age Range

eTable 3. Brain Growth Rate vs Gestational Age Between First and Second Scans

eTable 4. Distress Measures

eTable 5. Associations Between Maternal Distress Measures and Parental Socioeconomic Status Measures

eTable 6. Associations Between Fetal Brain Volume and Cortical Features and Parental Socioeconomic Status Measures

eTable 7. β Coefficients of Gestational Age for Associations Between Fetal Brain Volume and Parental Socioeconomic Status Measures

eTable 8. β Coefficients of Gestational Age for Associations Between Brain Lobe Volume and Cortical Features and Maternal Socioeconomic Measures

eTable 9. β Coefficients of Gestational Age for Associations Between Brain Lobe Volume and Cortial Features and Paternal Socioeconomic Measures

eTable 10. β Coefficients of Fetal Sex for Associations Between Fetal Brain Volume and Parental Socioeconomic Status Measures

eTable 11. β Coefficients of Fetal Sex for Associations Between Brain Lobe Volume and Cortical Features and Maternal Socioeconomic Status Measures

eTable 12. β Coefficients of Fetal Sex for Associations Between Brain Lobe Volume and Cortical Features and Paternal Socioeconomic Status Measures

eTable 13. Associations Between Fetal Brain Volumes and Parental Socioeconomic Status Measures

eTable 14. Associations Between Brain Lobe Volumes and Cortical Features and Maternal Socioeconomic Status Measures

© 2021 Lu YC et al. *JAMA Network Open.*

eTable 15. Associations Between Brain Lobe Volume and Cortical Features and Paternal Socioeconomic Status Measures

eFigure 1. Fetal Brain Magnetic Resonance Axial Plane Images and Reconstructed White Matter Surface at Different Gestational Ages

eFigure 2. Brain Tissue Segmentation

eFigure 3. Brain Parcellation

eFigure 4. Cortical Folding

eFigure 5. Subject Recruitment Flowchart

eFigure 6. Brain Tissue Volume vs Socioeconomic Status Score

eFigure 7. Brain Tissue Volume vs Gestational Age

eFigure 8. Cerebral Cortical Features vs Socioeconomic Status Score

eFigure 9. Cerebral Cortical Features vs Gestational Age

This supplemental material has been provided by the authors to give readers additional information about their work.

© 2021 Lu YC et al. *JAMA Network Open.*

eTable 1. Spearman Correlation Coefficients Among Socioeconomic Status Measures
All correlation coefficients were highly significant (*p*s<0.001).

	Maternal Education Level	Paternal Education Level	Maternal Occupation Level	Paternal Occupation Level	Maternal SES Score	Paternal SES Score
Maternal Education	1.00	0.71	0.70	0.74	0.89	0.75
Paternal Education		1.00	0.59	0.79	0.65	0.95
Maternal Occupation			1.00	0.74	0.91	0.65
Paternal Occupation				1.00	0.74	0.90
Maternal SES					1.00	0.71
Paternal SES						1.00

© 2021 Lu YC et al. *JAMA Network Open.*

eTable 2. Magnetic Resonance Imaging Scans obtained for Each Gestational Age Range Includes the median, interquartile range (IQR), minimum, and maximum GA in each GA range. GA: Gestational age.

GA Range	N of Scans	Median GA	GA IQR	Minimum GA	Maximum GA
24-25	3	24.4	0.5	24.0	24.7
25-26	6	25.6	0.3	25.0	25.9
26-27	6	26.4	0.6	26.0	26.7
27-28	12	27.6	0.4	27.0	27.9
28-29	14	28.3	0.3	28.1	28.7
29-30	13	29.3	0.5	29.1	29.9
30-31	8	30.2	0.4	30.1	30.7
31-32	13	31.4	0.4	31.0	31.9
32-33	8	32.4	0.2	32.0	32.7
33-34	16	33.6	0.4	33.0	33.9
34-35	16	34.6	0.5	34.0	34.9
35-36	20	35.3	0.5	35.0	35.9
36-37	21	36.4	0.6	36.0	36.9
37-38	15	37.3	0.4	37.0	37.9
38-39	10	38.3	0.4	38.0	38.9
39-40	3	39.1	0.2	39.1	39.4

© 2021 Lu YC et al. *JAMA Network Open.*

eTable 3. Brain Growth Rate vs Gestational Age Between First and Second Scans

The median and 25th and 75th percentiles (Q1 and Q3) of the slopes measuring the brain growth rate through the difference of brain volumes/cortical features vs. GA at MRI (weeks) between the first and second MRI scans for those (40 subjects) who underwent two MRIs.

Brain Tissue Volume (cm^3)					
	Low SES		High SES		
	Median	[Q1, Q3]	Median	[Q1, Q3]	p_{SES}
CGM	6.30	[6.11-7.54]	5.51	[5.13-6.08]	**0.01***
WM	8.02	[6.81-9.70]	9.59	[8.64-11.38]	**0.01***
Cerebellum	1.18	[1.12-1.38]	1.29	[1.06-1.39]	0.67
DGM	1.22	[1.15-1.27]	1.27	[1.20-1.41]	0.19
Brainstem	0.29	[0.26-0.31]	0.31	[0.28-0.32]	0.15
Whole Brain	18.29	[16.34-19.07]	18.73	[16.72-20.45]	0.18
Lobe Volume (cm^3)					
	Low SES		High SES		
	Median	[Q1, Q3]	Median	[Q1, Q3]	p_{SES}
Frontal	3.18	[2.84, 3.88]	4.06	[3.28, 4.57]	**0.02***
Parietal	2.08	[1.68, 2.54]	2.62	[2.12, 3.01]	**0.01***
Temporal	1.69	[1.62, 1.83]	1.90	[1.71, 2.14]	**0.04***
Occipital	0.78	[0.63, 0.99]	1.09	[0.94, 1.23]	**0.002***
LGI ($\times 10^{-3}$)					
	Low SES		High SES		
	Median	[Q1, Q3]	Median	[Q1, Q3]	p_{SES}
Frontal	41.3	[27.7, 54.0]	26.7	[22.3, 51.0]	0.15
Parietal	60.6	[50.8, 97.5]	51.7	[42.7, 65.1]	0.06
Temporal	50.5	[35.5, 75.0]	41.8	[38.0, 54.6]	0.49
Occipital	47.2	[36.8, 60.7]	50.1	[41.9, 58.3]	0.71
SDepth ($\times 10^{-3}$ mm)					
	Low SES		High SES		
	Median	[Q1, Q3]	Median	[Q1, Q3]	p_{SES}
Frontal	135	[66, 168]	97	[47, 163]	0.45
Parietal	236	[200, 275]	189	[159, 250]	0.07
Temporal	149	[104, 216]	124	[89, 166]	0.26
Occipital	154	[137, 179]	148	[132, 187]	0.67

© 2021 Lu YC et al. *JAMA Network Open.*

eTable 4. Distress Measures

The median and 25th and 75th percentiles (Q1 and Q3) of the distress measures. Chi-square test was used for comparing the number of scans. Linear mixed-effects models were used for assessing the associations between the maternal distress measures and parental SES (0: low SES; 1: high SES), adjusting for GA at MRI (weeks) and fetal sex (female: 0; male: 1). N: Number of scans. SSAI: Spielberger State Anxiety Inventory. STAI: Spielberger Trait Anxiety

	Low SES			High SES			p_{SES}
	N	Median	[Q1, Q3]	N	Median	[Q1, Q3]	
Low/High Distress	26/14			52/18			0.30
SSAI	43	27	[23, 32.75]	72	27	[23, 33]	0.54
STAI	42	28	[24, 35]	72	27.5	[24, 36]	0.72
PSS	42	10	[6, 15]	71	10	[6, 14]	0.97
EPDS	41	4	[1, 6]	72	3	[1, 6]	0.54

© 2021 Lu YC et al. *JAMA Network Open.*

eTable 5. Associations Between Maternal Distress Measures and Parental Socioeconomic Status Measures

The results of the linear mixed-effects models (LMM) for the associations between maternal distress measures and parental education/occupation/SES score, adjusting for GA at MRI (weeks) and fetal sex (female: 0; male: 1). GA: Gestational age. SSAI: Spielberger State Anxiety Inventory. STAI: Spielberger Trait Anxiety Inventory. PSS: Perceived Stress Scale. EPDS: Edinburgh Postnatal Depression Scale. None of the *p-values* were significant (*p*s>0.05).

	Maternal								
	Education			Occupation			SES Score		
	β_{M_Educ}	95% CI	p_{M_Educ}	β_{M_Occu}	95% CI	p_{M_Occu}	β_{M_SES}	95% CI	p_{M_SES}
SSAI	0.29	[-1.72, 2.29]	0.78	-0.85	[-2.41, 0.71]	0.28	-0.07	[-0.24, 0.11]	0.46
STAI	0.85	[-1.16, 2.86]	0.40	0.02	[-1.62, 1.65]	0.98	0.03	[-0.15, 0.21]	0.75
PSS	0.79	[-0.54, 2.11]	0.24	0.24	[-0.78, 1.26]	0.64	0.05	[-0.07, 0.16]	0.44
EPDS	0.30	[-0.53, 1.14]	0.47	0.24	[-0.43, 0.90]	0.48	0.03	[-0.04, 0.10]	0.42
	Paternal								
	Education			Occupation			SES Score		
	β_{P_Educ}	95% CI	p_{P_Educ}	β_{P_Occu}	95% CI	p_{P_Occu}	β_{P_SES}	95% CI	p_{P_SES}
STAI_S	0.11	[-1.48, 1.71]	0.89	-1.35	[-2.93, 0.23]	0.09	-0.08	[-0.24, 0.07]	0.28
STAI_T	0.60	[-0.99, 2.19]	0.46	-0.45	[-2.13, 1.22]	0.59	-0.005	[-0.17, 0.16]	0.95
PSS	0.48	[-0.57, 1.52]	0.37	-0.30	[-1.38, 0.77]	0.58	-0.001	[-0.11, 0.10]	0.98
EPDS	0.43	[-0.23, 1.10]	0.20	-0.06	[-0.75, 0.63]	0.86	0.01	[-0.05, 0.08]	0.70

© 2021 Lu YC et al. *JAMA Network Open.*

eTable 6. Associations Between Fetal Brain Volume and Cortical Features and Parental Socioeconomic Status Measures

The results of the linear mixed-effects models (LMM) for the associations between fetal brain volumes/cortical features and parental SES (0: low SES; 1: high SES), adjusting for GA at MRI (weeks) and fetal sex (female: 0; male: 1). LS Mean: Least squares mean. SE: Standard error. GA: Gestational age. CGM: Cortical gray matter. WM: White matter. DGM: Deep gray

Brain Tissue Volume (cm^3)					
	Low SES (LS Mean±SE)	High SES (LS Mean±SE)	β_{SES}	95% CI for β_{SES}	p_{SES}
CGM	70.8±5.0	68.1±5.1	-2.70	[-5.03, -0.37]	**0.02***
WM	107±8	113±8	5.87	[2.15, 9.60]	**0.002***
Cerebellum	10.5±0.8	10.9±0.9	0.41	[0.02, 0.80]	**0.04**
DGM	16.6±0.8	16.9±0.8	0.35	[-0.02, 0.73]	0.06
Brainstem	4.53±0.21	4.65±0.22	0.12	[0.02, 0.22]	**0.02***
Whole Brain	209±10	213±11	4.06	[-0.84, 8.96]	0.10
Lobe Volume (cm^3)					
	Low SES (LS Mean±SE)	High SES (LS Mean±SE)	β_{SES}	95% CI	p_{SES}
Frontal	42.2±3.4	44.1±3.5	1.93	[0.35, 3.52]	**0.02***
Parietal	26.4±2.4	28.1±2.4	1.70	[0.59, 2.81]	**0.003***
Temporal	20.5±1.4	21.6±1.4	1.07	[0.42, 1.72]	**0.001***
Occipital	9.7±1.1	10.2±1.1	0.44	[-0.06, 0.94]	0.09
LGI					
	Low SES (LS Mean±SE)	High SES (LS Mean±SE)	β_{SES} ($\times 10^{-3}$)	95% CI ($\times 10^{-3}$)	p_{SES}
Frontal	1.25±0.05	1.22±0.05	-29.0	[-51.7, -6.2]	**0.01***
Parietal	1.40±0.08	1.34±0.08	-58.9	[-94.6, -23.3]	**0.001***
Temporal	1.37±0.06	1.34±0.06	-31.3	[-59.7, -3.0]	**0.03***
Occipital	1.32±0.07	1.27±0.07	-47.6	[-79.6, -15.6]	**0.004***
SDepth (mm)					
	Low SES (LS Mean±SE)	High SES (LS Mean±SE)	β_{SES} ($\times 10^{-3}$)	95% CI ($\times 10^{-3}$)	p_{SES}
Frontal	1.53±0.20	1.47±0.20	-57.6	[-149.3, 34.2]	0.22
Parietal	2.25±0.27	2.00±0.28	-246.3	[-373.5, -119.1]	**<0.001***
Temporal	1.97±0.22	1.85±0.22	-120.0	[-220.9, -19.2]	**0.02***
Occipital	1.32±0.21	1.17±0.22	-157.2	[-255.8, -58.7]	**0.002***

© 2021 Lu YC et al. *JAMA Network Open.*

eTable 7. β coefficients of Gestational Age at Magnetic Resonance Imaging

The β coefficients of GA at MRI of the linear mixed-effects models (LMM) for the associations between fetal brain volumes (cm^3) and parental education/occupation/SES score, adjusting for GA at MRI (weeks) and fetal sex (female: 0; male: 1). GA: Gestational age. CGM: Cortical gray matter. WM: White matter. DGM: Deep gray matter. Bold: p<0.05. *: q<0.05.

	Maternal								
	Education			Occupation			SES Score		
	β_{GA}	95% CI	p_{GA}	β_{GA}	95% CI	p_{GA}	β_{GA}	95% CI	p_{GA}
CGM	5.90	[5.60, 6.19]	**<0.001***	5.89	[5.59, 6.19]	**<0.001***	5.89	[5.59, 6.19]	**<0.001***
WM	9.17	[8.69, 9.64]	**<0.001***	9.19	[8.72, 9.67]	**<0.001***	9.19	[8.72, 9.66]	**<0.001***
Cerebellum	1.21	[1.16, 1.26]	**<0.001***	1.21	[1.16, 1.26]	**<0.001***	1.21	[1.16, 1.26]	**<0.001***
DGM	1.23	[1.18, 1.28]	**<0.001***	1.23	[1.18, 1.28]	**<0.001***	1.23	[1.18, 1.28]	**<0.001***
Brainstem	0.29	[0.28, 0.30]	**<0.001***	0.29	[0.28, 0.30]	**<0.001***	0.29	[0.28, 0.30]	**<0.001***
Whole Brain	17.79	[17.17, 18.42]	**<0.001***	17.81	[17.19, 18.44]	**<0.001***	17.81	[17.18, 18.43]	**<0.001***
	Paternal								
	Education			Occupation			SES Score		
	β_{GA}	95% CI	p_{GA}	β_{GA}	95% CI	p_{GA}	β_{GA}	95% CI	p_{GA}
CGM	5.90	[5.60, 6.20]	**<0.001***	5.92	[5.62, 6.21]	**<0.001***	5.91	[5.61, 6.20]	**<0.001***
WM	9.18	[8.70, 9.65]	**<0.001***	9.13	[8.66, 9.61]	**<0.001***	9.15	[8.67, 9.62]	**<0.001***
Cerebellum	1.21	[1.16, 1.26]	**<0.001***	1.21	[1.15, 1.26]	**<0.001***	1.21	[1.16, 1.26]	**<0.001***
DGM	1.23	[1.18, 1.28]	**<0.001***	1.23	[1.18, 1.27]	**<0.001***	1.23	[1.18, 1.27]	**<0.001***
Brainstem	0.29	[0.28, 0.30]	**<0.001***	0.29	[0.28, 0.30]	**<0.001***	0.29	[0.28, 0.30]	**<0.001***
Whole Brain	17.80	[17.18, 18.42]	**<0.001***	17.77	[17.14, 18.40]	**<0.001***	17.78	[17.15, 18.40]	**<0.001***

© 2021 Lu YC et al. *JAMA Network Open.*

eTable 8. Associations Between Brain Lobe Volumes and Cortical Features and Maternal Socioeconomic Measures

The β coefficients of GA at MRI of the linear mixed-effects models (LMM) for the associations between the brain lobe volumes/cortical features and maternal education/occupation/SES score, adjusting for GA at MRI (weeks) and fetal sex (female: 0; male: 1). GA: Gestational age. LGI: Local gyrification index. SDepth: Sulcal depth. Bold:

	Maternal Education								
	Lobe Volume (cm^3)			LGI ($\times 10^{-3}$)			SDepth ($\times 10^{-3}$ mm)		
	β_{GA}	95% CI	p_{GA}	β_{GA}	95% CI	p_{GA}	β_{GA}	95% CI	p_{GA}
Frontal	3.59	[3.38, 3.79]	**<0.001***	35	[32, 38]	**<0.001***	104	[92, 116]	**<0.001***
Parietal	2.33	[2.19, 2.47]	**<0.001***	59	[54, 63]	**<0.001***	208	[192, 224]	**<0.001***
Temporal	1.80	[1.72, 1.88]	**<0.001***	48	[45, 52]	**<0.001***	132	[119, 144]	**<0.001***
Occipital	0.92	[0.86, 0.98]	**<0.001***	51	[47, 55]	**<0.001***	166	[154, 179]	**<0.001***
	Maternal Occupation								
	Lobe Volume (cm^3)			LGI ($\times 10^{-3}$)			SDepth ($\times 10^{-3}$ mm)		
	β_{GA}	95% CI	p_{GA}	β_{GA}	95% CI	p_{GA}	β_{GA}	95% CI	p_{GA}
Frontal	3.60	[3.39, 3.80]	**<0.001***	35	[32, 38]	**<0.001***	104	[92, 115]	**<0.001***
Parietal	2.33	[2.19, 2.48]	**<0.001***	58	[54, 63]	**<0.001***	207	[191, 223]	**<0.001***
Temporal	1.81	[1.72, 1.89]	**<0.001***	48	[45, 52]	**<0.001***	131	[118, 144]	**<0.001***
Occipital	0.92	[0.86, 0.99]	**<0.001***	50	[46, 54]	**<0.001***	165	[153, 178]	**<0.001***
	Maternal SES Score								
	Lobe Volume (cm^3)			LGI ($\times 10^{-3}$)			SDepth ($\times 10^{-3}$ mm)		
	β_{GA}	95% CI	p_{GA}	β_{GA}	95% CI	p_{GA}	β_{GA}	95% CI	p_{GA}
Frontal	3.59	[3.39, 3.80]	**<0.001***	35	[32, 38]	**<0.001***	104	[92, 115]	**<0.001***
Parietal	2.33	[2.19, 2.47]	**<0.001***	59	[54, 63]	**<0.001***	207	[191, 223]	**<0.001***
Temporal	1.81	[1.72, 1.89]	**<0.001***	48	[45, 52]	**<0.001***	131	[118, 144]	**<0.001***
Occipital	0.92	[0.86, 0.98]	**<0.001***	50	[46, 54]	**<0.001***	165	[153, 178]	**<0.001***

© 2021 Lu YC et al. *JAMA Network Open.*

eTable 9. β Coefficients Associations Between Brain Lobe Volumes and Cortial Features and Paternal Socioeconomic Measures

The β coefficients of GA at MRI of the linear mixed-effects models (LMM) for the associations between the brain lobe volumes/cortical features and paternal education/occupation/SES score, adjusting for GA at MRI (weeks) and fetal sex (female: 0; male: 1). GA: Gestational age. LGI: Local gyrification index. SDepth: Sulcal depth. Bold:

	Paternal Education								
	Lobe Volume (cm^3)			LGI (×10^{-3})			SDepth (×10^{-3} mm)		
	β_{GA}	95% CI	p_{GA}	β_{GA}	95% CI	p_{GA}	β_{GA}	95% CI	p_{GA}
Frontal	3.59	[3.39, 3.79]	**<0.001***	35	[32, 38]	**<0.001***	104	[92, 116]	**<0.001***
Parietal	2.33	[2.19, 2.47]	**<0.001***	59	[54, 63]	**<0.001***	207	[191, 224]	**<0.001***
Temporal	1.80	[1.72, 1.89]	**<0.001***	48	[45, 52]	**<0.001***	131	[118, 144]	**<0.001***
Occipital	0.92	[0.86, 0.98]	**<0.001***	50	[46, 54]	**<0.001***	165	[154, 178]	**<0.001***
	Paternal Occupation								
	Lobe Volume (cm^3)			LGI (×10^{-3})			SDepth (×10^{-3} mm)		
	β_{GA}	95% CI	p_{GA}	β_{GA}	95% CI	p_{GA}	β_{GA}	95% CI	p_{GA}
Frontal	3.58	[3.37, 3.78]	**<0.001***	35	[32, 38]	**<0.001***	104	[93, 116]	**<0.001***
Parietal	2.32	[2.18, 2.46]	**<0.001***	59	[55, 64]	**<0.001***	209	[193, 225]	**<0.001***
Temporal	1.79	[1.71, 1.88]	**<0.001***	49	[45, 52]	**<0.001***	132	[119, 145]	**<0.001***
Occipital	0.92	[0.85, 0.98]	**<0.001***	51	[47, 55]	**<0.001***	167	[155, 180]	**<0.001***
	Paternal SES Score								
	Lobe Volume (cm^3)			LGI (×10^{-3})			SDepth (×10^{-3} mm)		
	β_{GA}	95% CI	p_{GA}	β_{GA}	95% CI	p_{GA}	β_{GA}	95% CI	p_{GA}
Frontal	3.58	[3.38, 3.78]	**<0.001***	35	[32, 38]	**<0.001***	104	[92, 116]	**<0.001***
Parietal	2.32	[2.18, 2.46]	**<0.001***	59	[54, 63]	**<0.001***	209	[192, 225]	**<0.001***
Temporal	1.80	[1.72, 1.88]	**<0.001***	48	[45, 52]	**<0.001***	132	[119, 145]	**<0.001***
Occipital	0.92	[0.85, 0.98]	**<0.001***	51	[47, 55]	**<0.001***	167	[154, 179]	**<0.001***

© 2021 Lu YC et al. *JAMA Network Open.*

eTable 10. β Coefficients of Fetal Sex for Associations Between Fetal Brain Volume and Parental Socioeconomic Status Measures

The β coefficients of fetal sex of the linear mixed-effects models (LMM) for the associations between fetal brain volumes (cm^3) and parental education/occupation/SES score, adjusting for GA at MRI (weeks) and fetal sex (female: 0; male: 1). GA: Gestational age. CGM: Cortical gray matter. WM: White matter. DGM: Deep gray matter. Bold: p<0.05. *: q<0.05.

	Maternal								
	Education			Occupation			SES Score		
	β_{Gender}	95% CI	p_{Gender}	β_{Gender}	95% CI	p_{Gender}	β_{Gender}	95% CI	p_{Gender}
CGM	3.27	[0.96, 5.59]	**0.01***	3.31	[0.99, 5.64]	**0.01***	3.31	[0.99, 5.63]	**0.01***
WM	3.39	[-0.31, 7.08]	0.07	3.29	[-0.42, 7.00]	0.08	3.31	[-0.38, 7.00]	0.08
Cerebellum	0.002	[-0.39, 0.39]	0.99	-0.01	[-0.40, 0.39]	0.98	-0.005	[-0.39, 0.38]	0.98
DGM	0.35	[-0.02, 0.73]	0.06	0.35	[-0.03, 0.72]	0.07	0.35	[-0.03, 0.72]	0.07
Brainstem	0.10	[0.004, 0.20]	**0.04**	0.10	[0.002, 0.20]	**0.046**	0.10	[0.002, 0.20]	**0.04**
Whole Brain	7.12	[2.22, 12.01]	**0.005***	7.05	[2.15, 11.94]	**0.01***	7.06	[2.17, 11.95]	**0.005***
	Paternal								
	Education			Occupation			SES Score		
	β_{Gender}	95% CI	p_{Gender}	β_{Gender}	95% CI	p_{Gender}	β_{Gender}	95% CI	p_{Gender}
CGM	3.30	[0.96, 5.64]	**0.01***	3.15	[0.83, 5.47]	**0.01***	3.20	[0.88, 5.52]	**0.01***
WM	3.32	[-0.40, 7.03]	0.08	3.62	[-0.11, 7.35]	0.06	3.53	[-0.18, 7.25]	0.06
Cerebellum	-0.001	[-0.40, 0.39]	0.99	0.01	[-0.38, 0.41]	0.95	0.01	[-0.39, 0.41]	0.96
DGM	0.35	[-0.02, 0.72]	0.07	0.37	[-0.01, 0.74]	0.06	0.36	[-0.01, 0.74]	0.06
Brainstem	0.10	[0.002, 0.20]	**0.04**	0.11	[0.01, 0.21]	**0.04**	0.11	[0.01, 0.21]	**0.04**
Whole Brain	7.06	[2.17, 11.96]	**0.005***	7.25	[2.33, 12.17]	**0.004***	7.21	[2.30, 12.12]	**0.004***

© 2021 Lu YC et al. *JAMA Network Open.*

eTable 11. β coefficients of fetal sex for Associations Between Brain Lobe Volume and Cortical Features and Maternal Socioeconomic Status Measures

The β coefficients of fetal sex of the linear mixed-effects models (LMM) for the associations between the brain lobe volumes/cortical features and maternal education/occupation/SES score, adjusting for GA at MRI (weeks) and fetal sex (female: 0; male: 1). GA: Gestational age. LGI: Local gyrification index. SDepth: Sulcal depth. Bold: *p*<0.05. *: *q*<0.05.

	Maternal Education								
	Lobe Volume (cm^3)			LGI ($\times 10^{-3}$)			SDepth ($\times 10^{-3}$ mm)		
	β_{Gender}	95% CI	p_{Gender}	β_{Gender}	95% CI	p_{Gender}	β_{Gender}	95% CI	p_{Gender}
Frontal	1.09	[-0.49, 2.67]	0.17	21	[-2, 43]	0.08	39	[-53, 131]	0.41
Parietal	0.96	[-0.13, 2.06]	0.08	30	[-5, 65]	0.10	77	[-49, 204]	0.23
Temporal	0.80	[0.16, 1.44]	**0.01**	10	[-18, 38]	0.47	27	[-73, 127]	0.60
Occipital	0.51	[0.01, 1.01]	**0.04**	9	[-23, 40]	0.58	11	[-85, 107]	0.83
	Maternal Occupation								
	Lobe Volume (cm^3)			LGI ($\times 10^{-3}$)			SDepth ($\times 10^{-3}$ mm)		
	β_{Gender}	95% CI	p_{Gender}	β_{Gender}	95% CI	p_{Gender}	β_{Gender}	95% CI	p_{Gender}
Frontal	1.06	[-0.52, 2.64]	0.19	21	[-2, 44]	0.07	40	[-52, 131]	0.39
Parietal	0.93	[-0.17, 2.03]	0.10	31	[-4, 66]	0.09	81	[-45, 208]	0.21
Temporal	0.78	[0.14, 1.43]	**0.02**	11	[-17, 39]	0.44	29	[-71, 129]	0.57
Occipital	0.50	[0.01, 1.00]	**0.048**	10	[-22, 41]	0.55	14	[-83, 111]	0.78
	Maternal SES Score								
	Lobe Volume (cm^3)			LGI ($\times 10^{-3}$)			SDepth ($\times 10^{-3}$ mm)		
	β_{Gender}	95% CI	p_{Gender}	β_{Gender}	95% CI	p_{Gender}	β_{Gender}	95% CI	p_{Gender}
Frontal	1.07	[-0.51, 2.65]	0.18	21	[-2, 44]	0.07	39	[-52, 131]	0.40
Parietal	0.94	[-0.16, 2.03]	0.09	31	[-4, 66]	0.09	81	[-45, 206]	0.21
Temporal	0.79	[0.14, 1.43]	**0.02**	11	[-17, 39]	0.45	29	[-71, 129]	0.57
Occipital	0.51	[0.01, 1.00]	**0.047**	9	[-22, 41]	0.56	13	[-83, 109]	0.79

© 2021 Lu YC et al. *JAMA Network Open.*

eTable 12. β Coefficients of Fetal Sex for Associations Between Brain Lobe Volume and Cortical Features and Paternal Socioeconomic Status Measures

The β coefficients of fetal sex of the linear mixed-effects models (LMM) for the associations between the brain lobe volumes/cortical features and paternal education/occupation/SES score, adjusting for GA at MRI (weeks) and fetal sex (female: 0; male: 1). GA: Gestational age. LGI: Local gyrification index. SDepth: Sulcal depth. Bold: p<0.05. *: q<0.05.

	Paternal Education								
	Lobe Volume (cm^3)			LGI ($\times 10^{-3}$)			SDepth ($\times 10^{-3}$ mm)		
	β_{Gender}	95% CI	p_{Gender}	β_{Gender}	95% CI	p_{Gender}	β_{Gender}	95% CI	p_{Gender}
Frontal	1.07	[-0.51, 2.66]	0.18	21	[-2, 44]	0.07	39	[-53, 131]	0.40
Parietal	0.94	[-0.16, 2.05]	0.09	31	[-5, 66]	0.09	80	[-48, 208]	0.22
Temporal	0.79	[0.14, 1.43]	**0.02**	11	[-18, 39]	0.46	28	[-72, 129]	0.58
Occipital	0.51	[0.01, 1.00]	**0.047**	9	[-22, 41]	0.56	13	[-84, 110]	0.79
	Paternal Occupation								
	Lobe Volume (cm^3)			LGI ($\times 10^{-3}$)			SDepth ($\times 10^{-3}$ mm)		
	β_{Gender}	95% CI	p_{Gender}	β_{Gender}	95% CI	p_{Gender}	β_{Gender}	95% CI	p_{Gender}
Frontal	1.16	[-0.43, 2.75]	0.15	20	[-3, 43]	0.09	37	[-55, 129]	0.43
Parietal	1.04	[-0.07, 2.14]	0.07	27	[-8, 63]	0.13	67	[-60, 195]	0.30
Temporal	0.85	[0.21, 1.50]	**0.01***	9	[-19, 37]	0.54	21	[-79, 121]	0.68
Occipital	0.53	[0.03, 1.03]	**0.04**	7	[-25, 38]	0.68	3	[-94, 101]	0.95
	Paternal SES Score								
	Lobe Volume (cm^3)			LGI ($\times 10^{-3}$)			SDepth ($\times 10^{-3}$ mm)		
	β_{Gender}	95% CI	p_{Gender}	β_{Gender}	95% CI	p_{Gender}	β_{Gender}	95% CI	p_{Gender}
Frontal	1.14	[-0.45, 2.73]	0.16	20	[-3, 43]	0.09	38	[-54, 129]	0.42
Parietal	1.01	[-0.09, 2.11]	0.07	28	[-7, 64]	0.12	71	[-56, 198]	0.27
Temporal	0.83	[0.19, 1.47]	**0.01***	9	[-19, 38]	0.51	23	[-77, 123]	0.65
Occipital	0.52	[0.02, 1.02]	**0.04**	7	[-24, 39]	0.65	6	[-91, 103]	0.91

© 2021 Lu YC et al. *JAMA Network Open.*

eTable 13. Associations Between Fetal Brain Volumes and Parental Socioeconomic Status Measures

The results of the linear mixed-effects models (LMM) for the associations between fetal brain volumes (cm^3) and parental education/occupation/SES score, adjusting for GA at MRI (weeks), fetal sex (female: 0; male: 1), and maternal distress (low: 0; high: 1). GA: Gestational age. CGM: Cortical gray matter. WM: White matter. DGM: Deep gray matter. Bold: $p<0.05$, *: $q<0.05$

	Maternal								
	Education			Occupation			SES Score		
	β_{M_Educ}	95% CI	p_{M_Educ}	β_{M_Occu}	95% CI	p_{M_Occu}	β_{M_SES}	95% CI	p_{M_SES}
CGM	0.03	[-1.71, 1.76]	0.97	0.38	[-1.05, 1.82]	0.60	0.03	[-0.13, 0.19]	0.67
WM	5.04	[2.08, 8.00]	**0.001***	2.72	[0.20, 5.24]	**0.03**	0.41	[0.13, 0.69]	**0.004***
Cerebellum	0.20	[-0.09, 0.48]	0.17	0.05	[-0.19, 0.29]	0.69	0.01	[-0.02, 0.04]	0.41
DGM	0.38	[0.10, 0.67]	**0.01***	0.22	[-0.02, 0.46]	0.07	0.03	[0.01, 0.06]	**0.02***
Brainstem	0.12	[0.04, 0.20]	**0.003***	0.03	[-0.03, 0.10]	0.31	0.01	[-0.0002, 0.01]	0.06
Whole Brain	5.77	[1.93, 9.60]	**0.004***	3.40	[0.17, 6.64]	**0.04**	0.50	[0.14, 0.85]	**0.01***
	Paternal								
	Education			Occupation			SES Score		
	β_{P_Educ}	95% CI	p_{P_Educ}	β_{P_Occu}	95% CI	p_{P_Occu}	β_{P_SES}	95% CI	p_{P_SES}
CGM	0.13	[-1.31, 1.56]	0.86	0.30	[-1.21, 1.81]	0.70	0.02	[-0.12, 0.17]	0.74
WM	3.25	[0.76, 5.75]	**0.01**	3.15	[0.50, 5.79]	**0.02**	0.34	[0.08, 0.59]	**0.01**
Cerebellum	0.14	[-0.09, 0.38]	0.23	0.02	[-0.23, 0.27]	0.85	0.01	[-0.02, 0.03]	0.54
DGM	0.19	[-0.05, 0.43]	0.12	0.14	[-0.11, 0.40]	0.27	0.02	[-0.01, 0.04]	0.17
Brainstem	0.06	[-0.003, 0.13]	0.06	0.07	[0.004, 0.14]	**0.04**	0.01	[0.001, 0.01]	**0.03**
Whole Brain	3.78	[0.56, 7.00]	**0.02**	3.68	[0.28, 7.09]	**0.03**	0.39	[0.07, 0.72]	**0.02**

© 2021 Lu YC et al. *JAMA Network Open.*

eTable 14. Associations Between Brain Lobe Volumes and Cortical Features and Maternal Socioeconomic Status Measures

The results of the linear mixed-effects models (LMM) for the associations between the brain lobe volumes/cortical features and maternal education/occupation/SES score, adjusting for GA at MRI (weeks), fetal sex (female: 0; male: 1), and maternal distress (low: 0; high: 1). GA: Gestational age. LGI: Local gyrification index. SDepth: Sulcal depth. Bold: p<0.05. *: q<0.05.

	Maternal Education								
	Lobe Volume (cm^3)			LGI ($\times10^{-3}$)			SDepth ($\times10^{-3}$ mm)		
	β_{M_LobeV}	95% CI	p_{M_LobeV}	β_{M_LGI}	95% CI	p_{M_LGI}	β_{M_SDepth}	95% CI	p_{M_SDepth}
Frontal	1.89	[0.56, 3.22]	**0.01***	-16	[-32, 1]	0.07	-43	[-113, 28]	0.23
Parietal	1.27	[0.42, 2.13]	**0.004***	-27	[-52, -3]	**0.03**	-105	[-196, -13]	**0.03**
Temporal	0.84	[0.33, 1.35]	**0.002***	-5	[-26, 16]	0.64	-28	[-103, 47]	0.46
Occipital	0.50	[0.14, 0.87]	**0.01***	-19	[-41, 3]	0.09	-40	[-108, 28]	0.25
	Maternal Occupation								
	Lobe Volume (cm^3)			LGI ($\times10^{-3}$)			SDepth ($\times10^{-3}$ mm)		
	β_{M_LobeV}	95% CI	p_{M_LobeV}	β_{M_LGI}	95% CI	p_{M_LGI}	β_{M_SDepth}	95% CI	p_{M_SDepth}
Frontal	0.92	[-0.21, 2.04]	0.11	-7	[-21, 7]	0.32	3	[-55, 62]	0.91
Parietal	0.63	[-0.09, 1.36]	0.09	-19	[-39, 2]	0.07	-50	[-126, 27]	0.20
Temporal	0.43	[-0.01, 0.86]	0.06	-6	[-24, 11]	0.48	-29	[-91, 34]	0.36
Occipital	0.28	[-0.03, 0.59]	0.08	-13	[-32, 5]	0.16	-22	[-78, 34]	0.44
	Maternal SES Score								
	Lobe Volume (cm^3)			LGI ($\times10^{-3}$)			SDepth ($\times10^{-3}$ mm)		
	β_{M_LobeV}	95% CI	p_{M_LobeV}	β_{M_LGI}	95% CI	p_{M_LGI}	β_{M_SDepth}	95% CI	p_{M_SDepth}
Frontal	0.14	[0.02, 0.27]	**0.02***	-1.2	[-2.7, 0.4]	0.15	-1.2	[-7.7, 5.4]	0.72
Parietal	0.10	[0.02, 0.18]	**0.02***	-2.6	[-4.8, -0.3]	**0.03**	-7.9	[-16.5, 0.6]	0.07
Temporal	0.07	[0.02, 0.11]	**0.01***	-0.7	[-2.7, 1.2]	0.47	-3.5	[-10.4, 3.5]	0.32
Occipital	0.04	[0.01, 0.08]	**0.02***	-1.8	[-3.9, 0.3]	0.08	-3.3	[-9.6, 3.0]	0.30

© 2021 Lu YC et al. *JAMA Network Open.*

eTable 15. Associations Between Brain lobe Volumes and Cortical Features and Paternal Socioeconomic Status Measures

The results of the linear mixed-effects models (LMM) for the associations between the brain lobe volumes/cortical features and paternal education/occupation/SES score, adjusting for GA at MRI (weeks), fetal sex (female: 0; male: 1), and maternal distress (low: 0; high: 1). GA: Gestational age. LGI: Local gyrification index. SDepth: Sulcal depth. Bold: p<0.05. *: q<0.05.

	Paternal Education								
	Lobe Volume (cm^3)			LGI (×10^{-3})			SDepth (×10^{-3} mm)		
	β_{P_LobeV}	95% CI	p_{P_LobeV}	β_{P_LGI}	95% CI	p_{P_LGI}	β_{P_SDepth}	95% CI	p_{P_SDepth}
Frontal	1.29	[0.18, 2.40]	**0.02***	-9	[-23, 5]	0.19	-15	[-73, 44]	0.62
Parietal	0.60	[-0.13, 1.33]	0.11	-17	[-37, 4]	0.11	-48	[-125, 29]	0.22
Temporal	0.53	[0.09, 0.96]	**0.02***	-2	[-20, 16]	0.81	-20	[-82, 43]	0.53
Occipital	0.33	[0.02, 0.63]	**0.04**	-11	[-29, 8]	0.25	-37	[-93, 19]	0.20
	Paternal Occupation								
	Lobe Volume (cm^3)			LGI (×10^{-3})			SDepth (×10^{-3} mm)		
	β_{P_LobeV}	95% CI	p_{P_LobeV}	β_{P_LGI}	95% CI	p_{P_LGI}	β_{P_SDepth}	95% CI	p_{P_SDepth}
Frontal	1.11	[-0.07, 2.28]	0.07	-14	[-29, 1]	0.06	-48	[-109, 13]	0.12
Parietal	0.75	[-0.02, 1.51]	0.055	-21	[-42, 0]	0.06	-59	[-139, 22]	0.15
Temporal	0.60	[0.15, 1.06]	**0.01***	-9	[-28, 9]	0.33	-31	[-97, 34]	0.35
Occipital	0.32	[-0.01, 0.64]	0.055	-17	[-37, 2]	0.08	-41	[-100, 18]	0.17
	Paternal SES Score								
	Lobe Volume (cm^3)			LGI (×10^{-3})			SDepth (×10^{-3} mm)		
	β_{P_LobeV}	95% CI	p_{P_LobeV}	β_{P_LGI}	95% CI	p_{P_LGI}	β_{P_SDepth}	95% CI	p_{P_SDepth}
Frontal	0.12	[0.01, 0.24]	**0.03***	-1.3	[-2.7, 0.1]	0.07	-3.7	[-9.6, 2.2]	0.21
Parietal	0.07	[-0.0003, 0.15]	0.051	-2.0	[-4.1, 0.0]	0.053	-5.8	[-13.5, 2.0]	0.15
Temporal	0.06	[0.02, 0.10]	**0.01***	-0.7	[-2.5, 1.1]	0.45	-2.8	[-9.1, 3.5]	0.38
Occipital	0.03	[0.003, 0.07]	**0.03***	-1.6	[-3.4, 0.3]	0.10	-4.2	[-9.8, 1.5]	0.15

© 2021 Lu YC et al. *JAMA Network Open.*

eFigure 1. Fetal Brain Magneteic Resonance Axial Plane Images and Reconstructed White Matter Surface at Different Gestational Ages

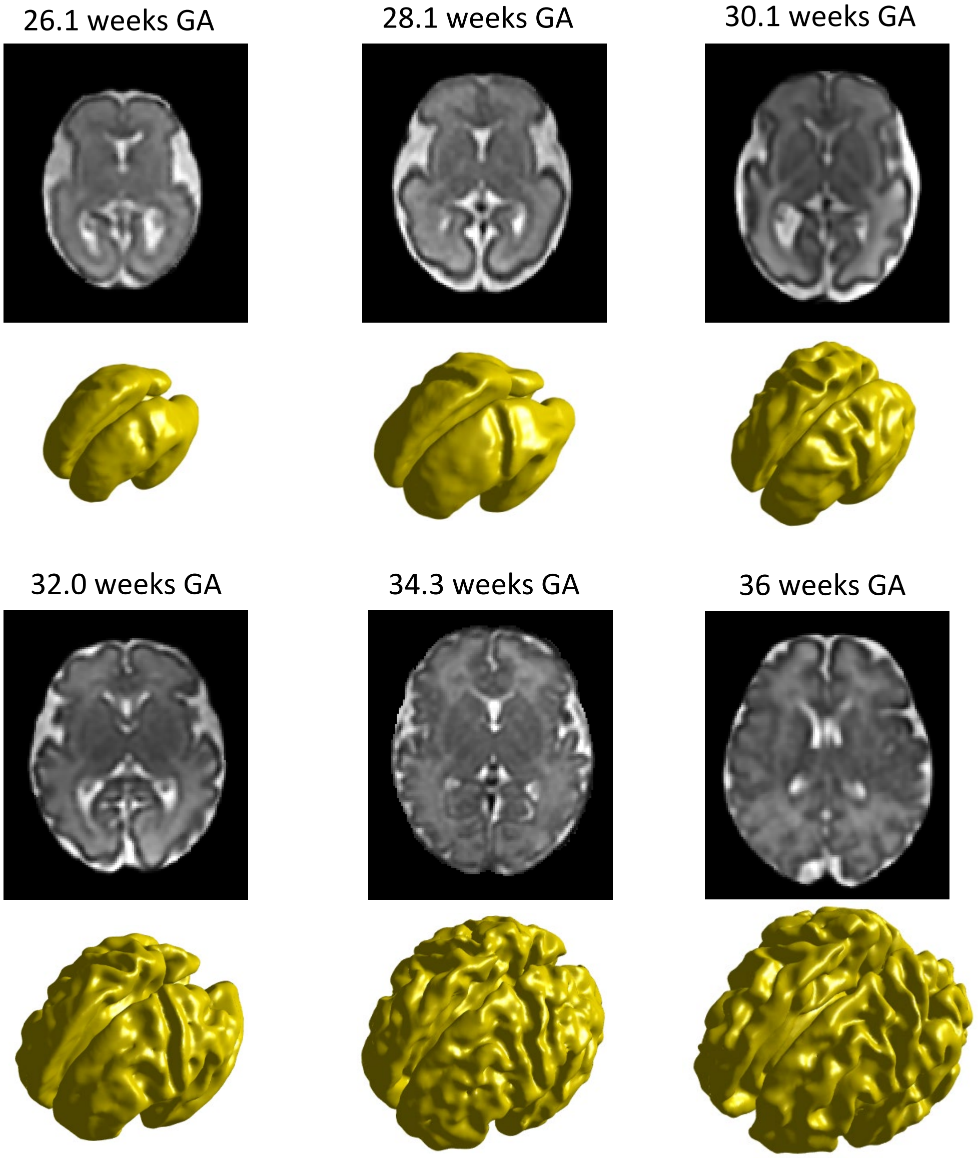

© 2021 Lu YC et al. *JAMA Network Open.*

eFigure 2. Brain Tissue Segmentation
Illustration of Brain Tissue Segmentation by Draw-EM Algorithm and Manual Correction
(a) Axial plane; (b) Coronal plane; (c) Sagittal plane.

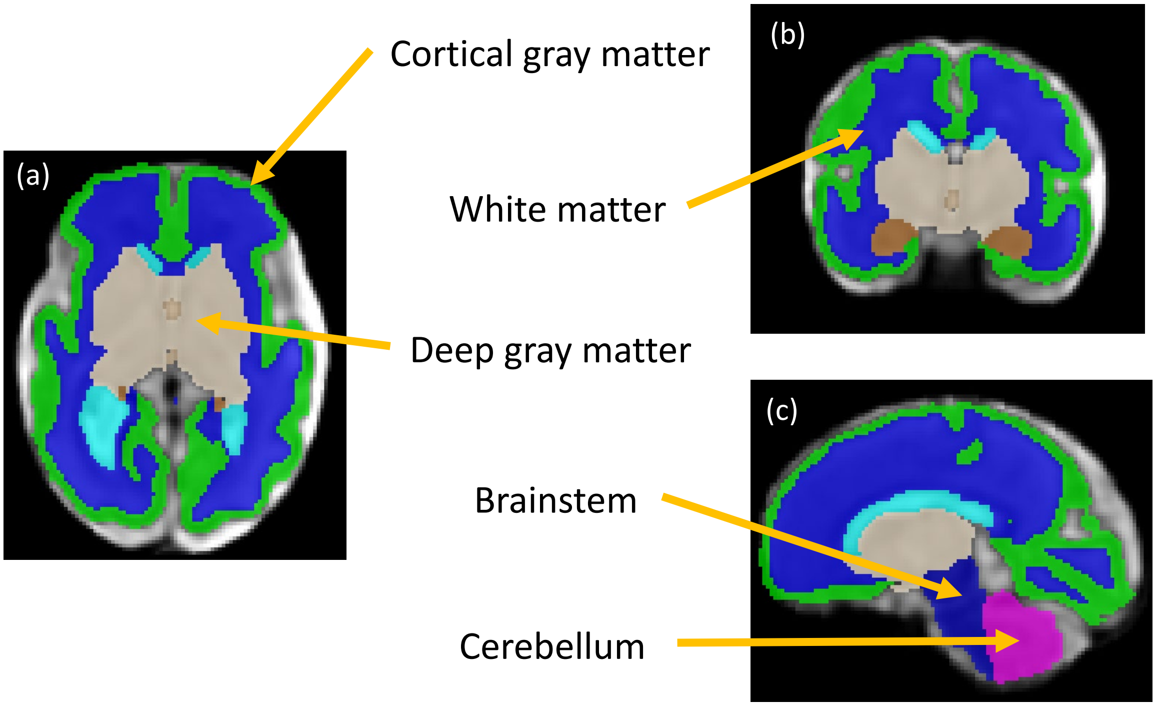

© 2021 Lu YC et al. *JAMA Network Open.*

eFigure 3. Brain Parcellation

Illustration of brain parcellation by Draw-EM algorithm and manual correction. (a) Lateral view; (b) Medial view.

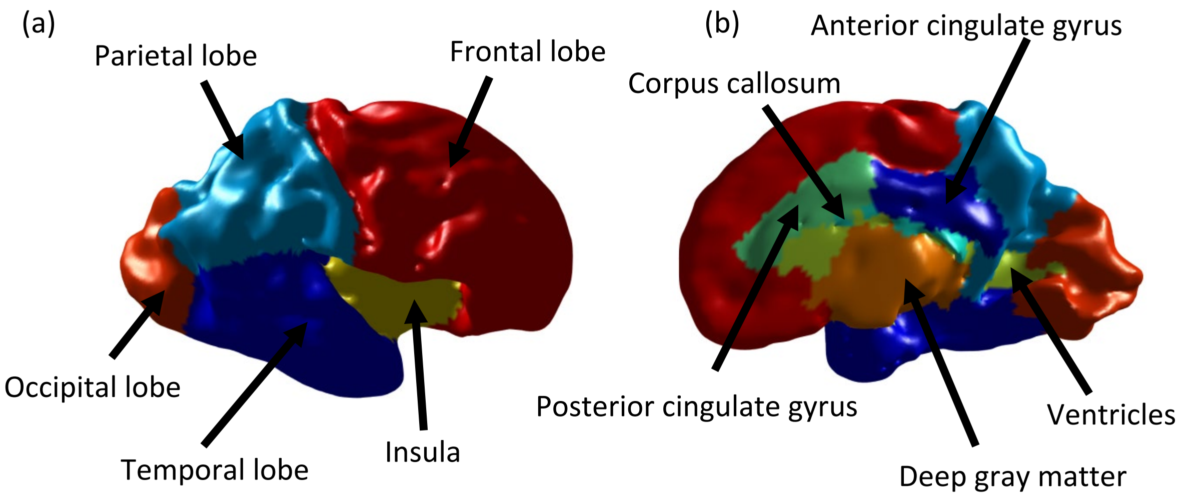

© 2021 Lu YC et al. *JAMA Network Open.*

eFigure 4. Cortical Folding

Illustration of cortical folding features on white matter surfaces. (a) Local gyrification index; (b) Sulcal depth.

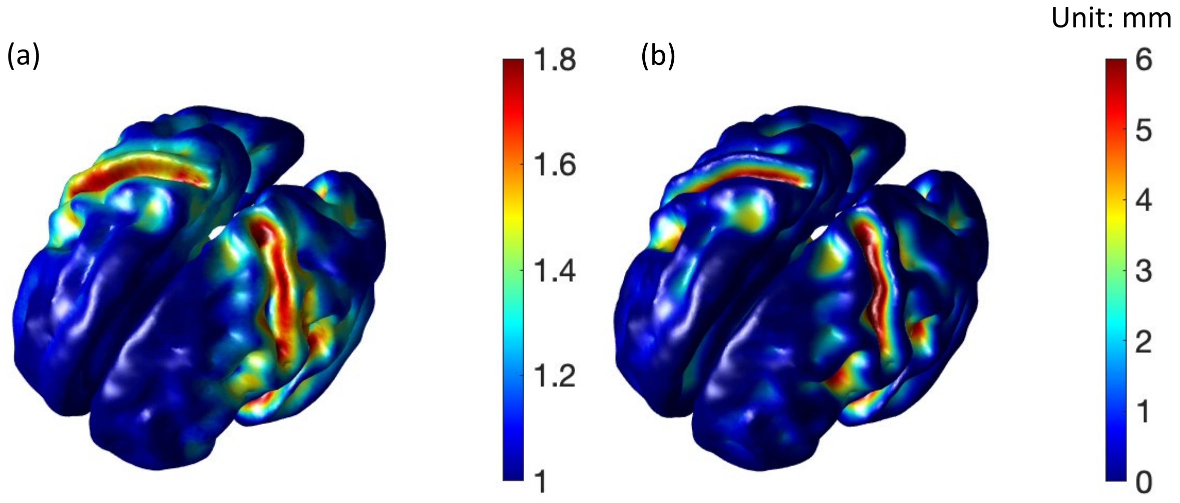

© 2021 Lu YC et al. *JAMA Network Open.*

eFigure 5. Subject Recruitment Flowchart

Flow diagram summarizing our subject recruitment in this study. Eligible women were recruited from community maternal fetal medicine offices and referred by their obstetrics providers. Once screened for eligibility, mothers were invited to join the study by a fetal cardiologist. The study team spoke to potential participants alongside the fetal cardiologists and would follow up with those interested in participation. Written informed consent was obtained from all participants before completing study procedures.

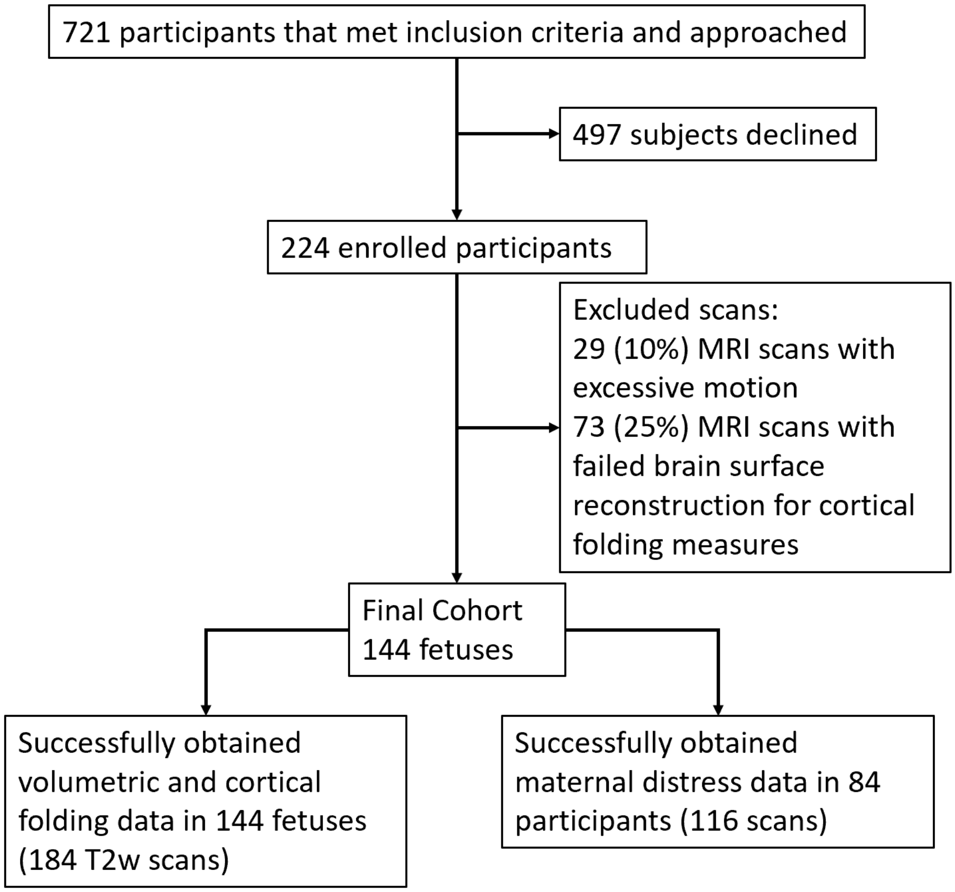

© 2021 Lu YC et al. *JAMA Network Open.*

eFigure 6. Brain Tissue Volume vs Socioeconomic Status Score

Scatter plots of brain tissue volumes vs. SES scores. (a) Cortical gray matter (CGM); (b) White matter (WM); (c) Cerebellum; (d) Deep gray matter (DGM); (e) Brainstem; (f) Whole brain. The straight lines are the linear fit of the data by parental sex. The *p-values* are corresponding to the p_{M_SES} and p_{P_SES} shown in Table 3. Bold *p*: *p*<0.05.

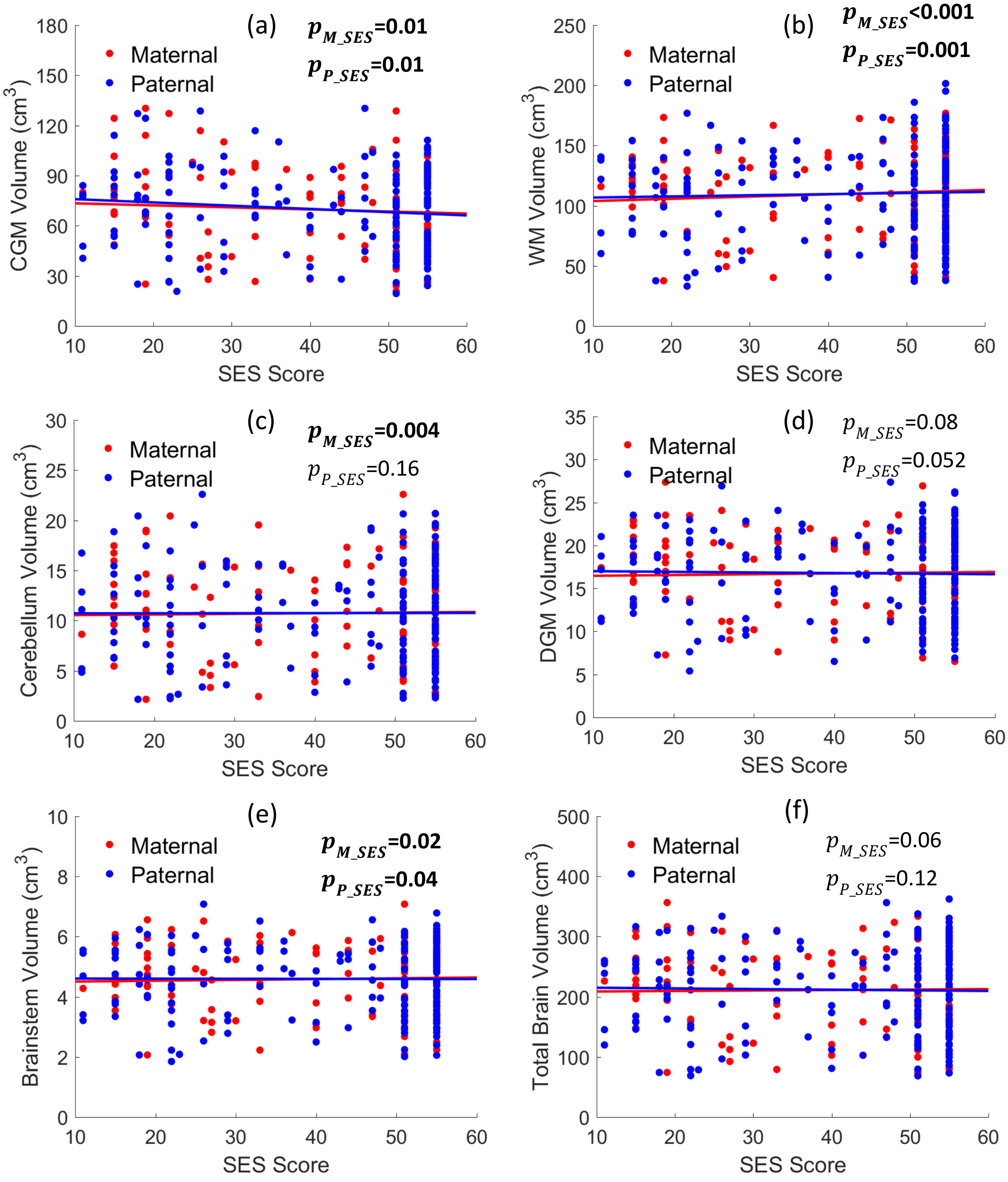

© 2021 Lu YC et al. *JAMA Network Open.*

eFigure 7. Brain Tissue Volume vs Gestational Age

Scatter plots of brain tissue volumes vs. gestational age. (a) Cortical gray matter (CGM); (b) White matter (WM); (c) Cerebellum; (d) Deep gray matter (DGM); (e) Brainstem; (f) Whole brain. The straight lines are the linear fit of the data by parental SES. The *p-values* are corresponding to p_{SES} shown in eTable 6. Bold *p*: *p*<0.05.

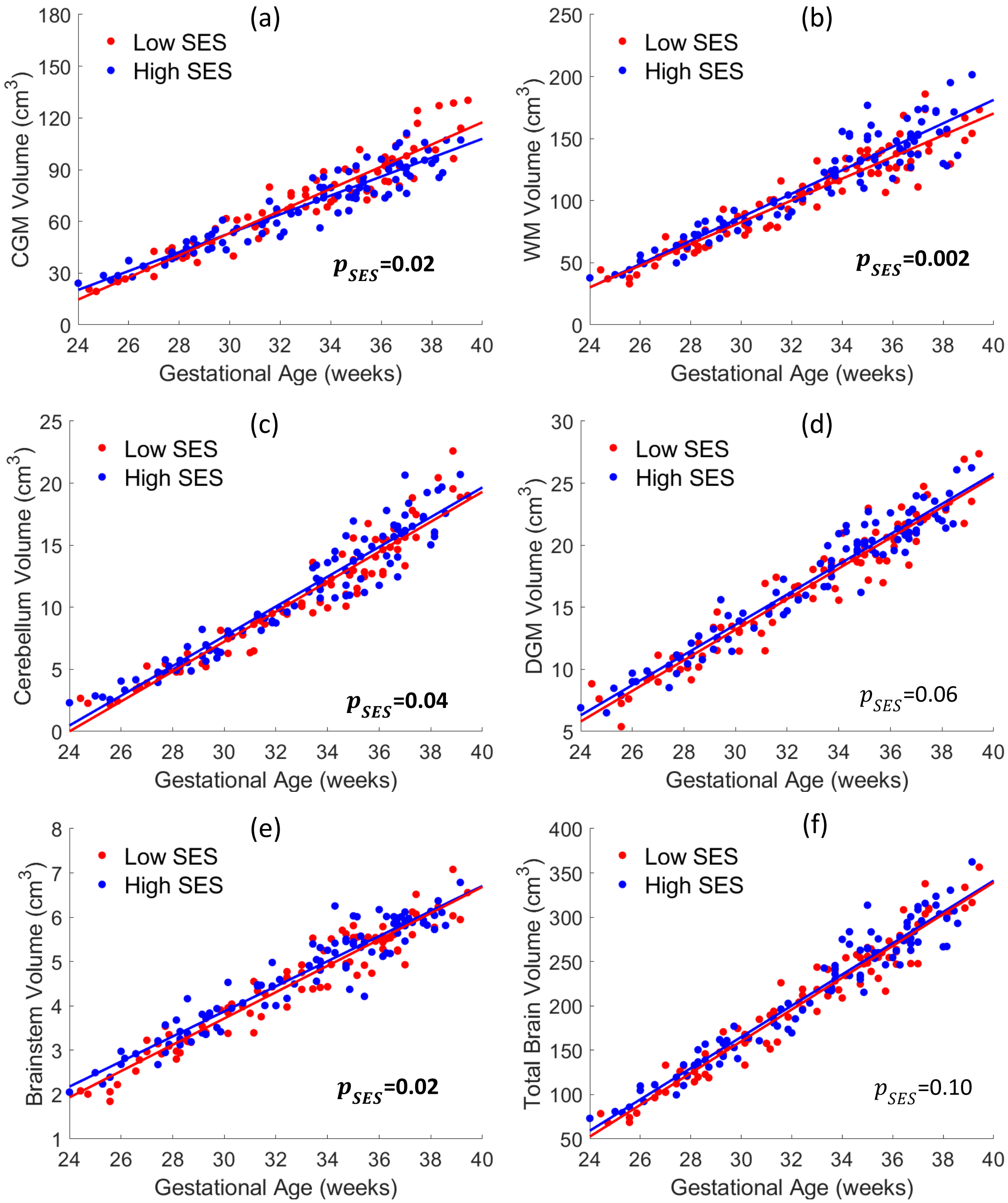

© 2021 Lu YC et al. *JAMA Network Open.*

eFigure 8. Cerebral Cortical Features vs Socioeconomic Status Score

Scatter plots of brain lobe volume/LGI/SDepth vs. SES scores. (a)-(c): Frontal lobe; (d)-(f) Parietal lobe; (g)-(i): Temporal lobe; (j)-(l): Occipital lobe. The straight lines are the linear fit of the data by parental sex. The *p-values* are corresponding to the maternal p_{M_LobeV}, p_{M_LGI}, and p_{M_SDepth} and paternal p_{P_LobeV}, p_{P_LGI}, and p_{P_SDepth} shown in Table 4 and 5. LGI: Local gyrification index. SDepth: Sulcal depth. Bold *p*: *p*<0.05.

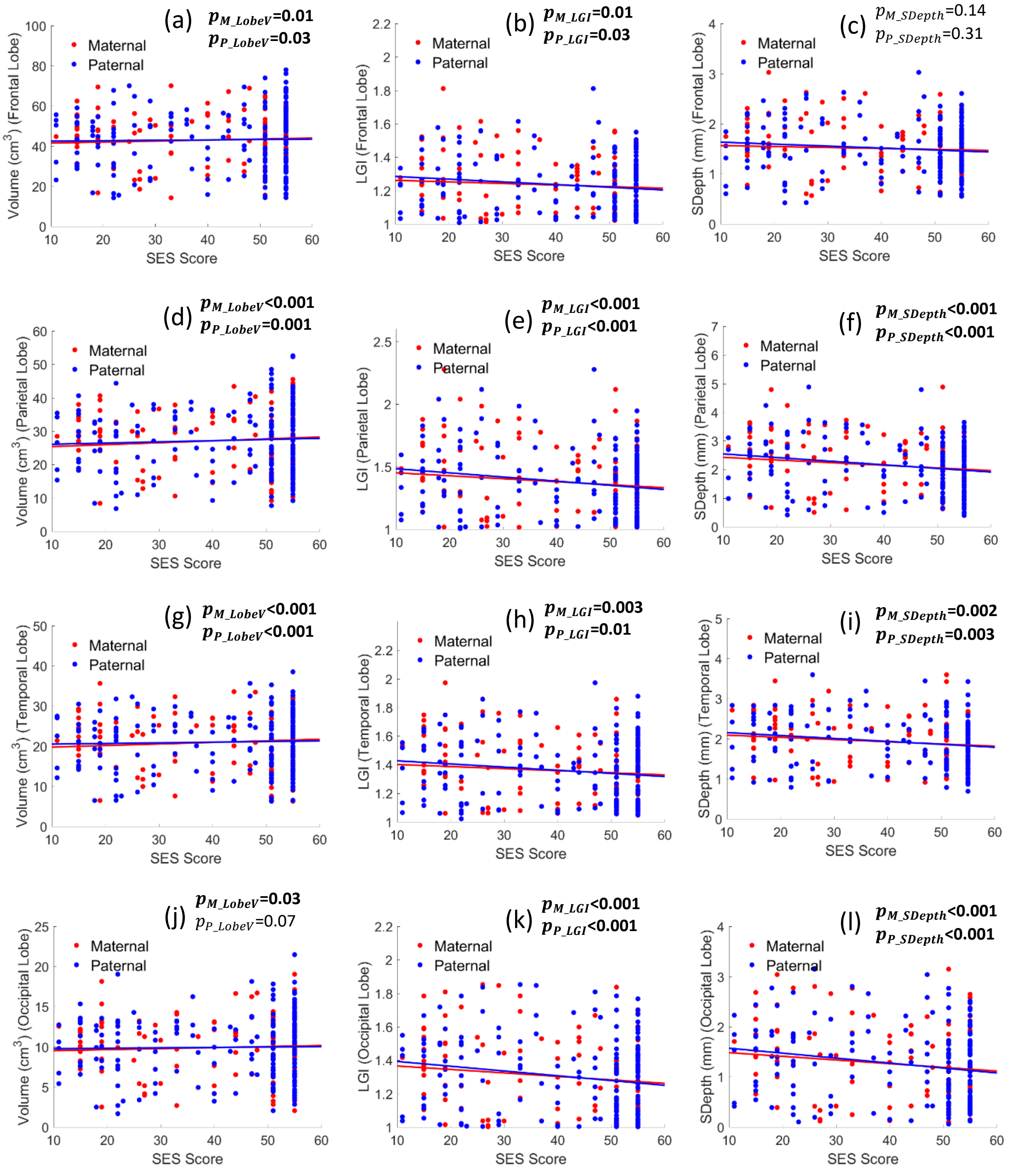

© 2021 Lu YC et al. *JAMA Network Open.*

eFigure 9. Cerebral Cortical Features vs Gestational Age

Scatter plots of brain lobe volume/LGI/SDepth vs. gestational age. (a)-(c): Frontal lobe; (d)-(f) Parietal lobe; (g)-(i): Temporal lobe; (j)-(l): Occipital lobe. The straight lines are the linear fit of the data by parental SES. The *p-values* are corresponding to p_{SES} shown in eTable 6. LGI: Local gyrification index. SDepth: Sulcal depth. Bold *p*: *p*<0.05.

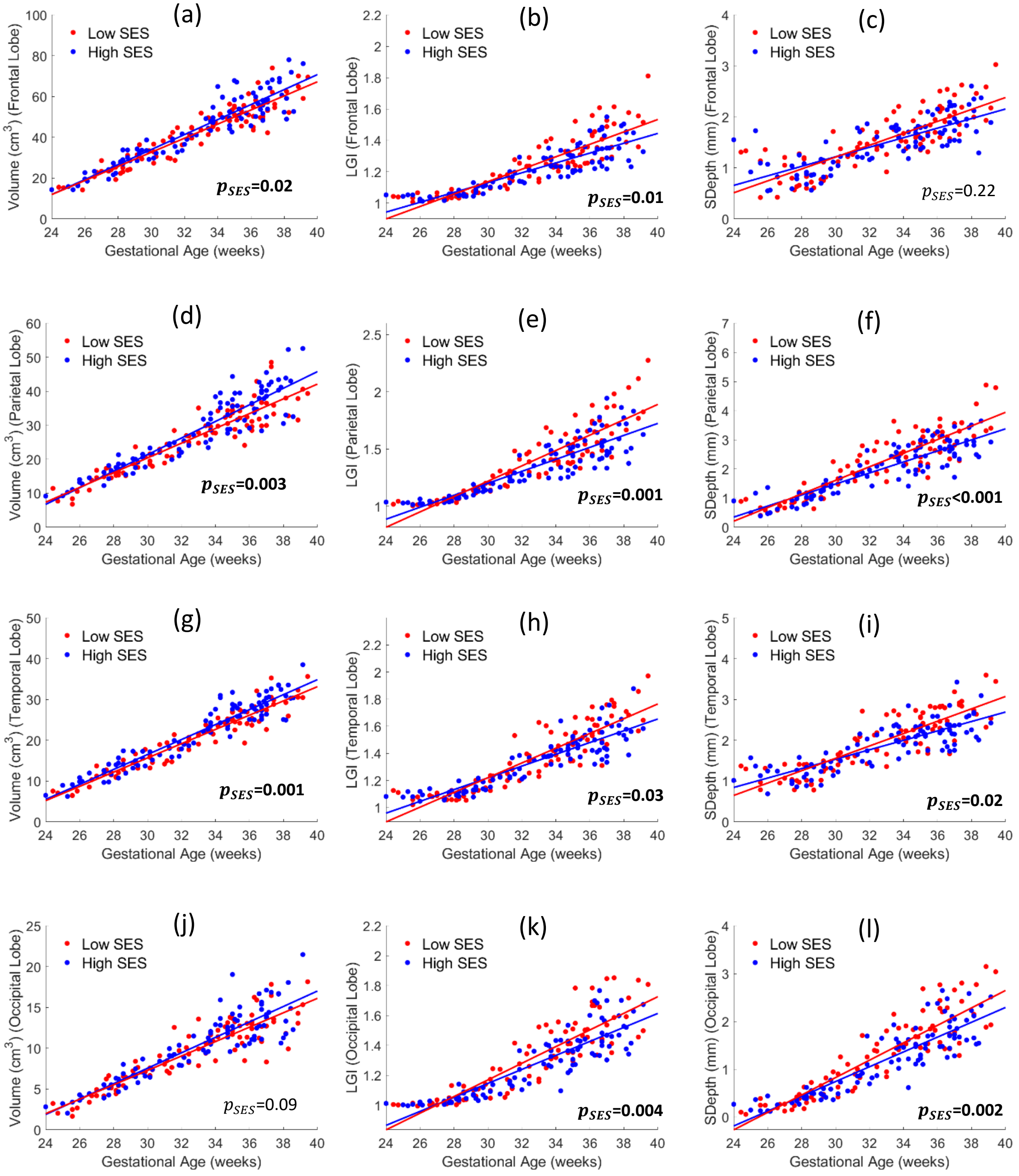

© 2021 Lu YC et al. *JAMA Network Open.*
